# Effects of Antipsychotics on the Hypothalamus–Pituitary–Adrenal Axis in a Phencyclidine Animal Model of Schizophrenia

**DOI:** 10.3390/cells13171425

**Published:** 2024-08-26

**Authors:** Tatjana Nikolić, Milica Velimirović Bogosavljević, Tihomir Stojković, Selma Kanazir, Nataša Lončarević-Vasiljković, Nevena V. Radonjić, Jelena Popić, Nataša Petronijević

**Affiliations:** 1 Institute of Medical and Clinical Biochemistry, Faculty of Medicine, University of Belgrade, 11000 Belgrade, Serbia; tatjana.nikolic@med.bg.ac.rs (T.N.); milica.velimirovic@med.bg.ac.rs (M.V.B.);; 2 Department of Neurobiology, Institute for Biological Research, University of Belgrade, 11000 Belgrade, Serbia; selkan@ibiss.bg.ac.rs; 3 iNOVA4Health, NOVA Medical School|Faculdade Ciências Médicas, NMS|FCM, Universidade Nova de Lisboa, Campo dos Mártires da Pátria, 1169-056 Lisbon, Portugal; natasa.loncarevic@nms.unl.pt; 4 Department of Psychiatry and Behavioral Sciences, SUNY Upstate Medical University, Syracuse, NY 13210, USA; radonjin@upstate.edu; 5 Department of Neurology and Neurosurgery, McGill University, Montreal, QC H3A 0G4, Canada; jelena.popic@mcgill.ca

**Keywords:** animal model, antipsychotic, clozapine, haloperidol, phencyclidine, schizophrenia, HPA axis, corticosterone

## Abstract

Schizophrenia (SCH) is a mental disorder that requires long-term antipsychotic treatment. SCH patients are thought to have an increased sensitivity to stress. The dysregulation of the hypothalamic–pituitary–adrenal (HPA) axis, observed in SCH, could include altered levels of glucocorticoids, glucocorticoid receptors (GRs), and associated proteins. The perinatal administration of phencyclidine (PCP) to rodents represents an animal model of SCH. This study investigated the effects of perinatal PCP exposure and subsequent haloperidol/clozapine treatment on corticosterone levels measured by ELISA and the expression of GR-related proteins (GR, pGR, HSP70, HSP90, FKBP51, and 11β-Hydroxysteroid dehydrogenase-11β-HSD) determined by Western blot, in different brain regions of adult rats. Six groups of male rats were treated on the 2nd, 6th, 9th, and 12th postnatal days (PN), with either PCP or saline. Subsequently, one saline and one PCP group received haloperidol/clozapine from PN day 35 to PN day 100. The results showed altered GR sensitivity in the rat brain after PCP exposure, which decreased after haloperidol/clozapine treatment. These findings highlight disturbances in the HPA axis in a PCP-induced model of SCH and the potential protective effects of antipsychotics. To the best of our knowledge, this is the first study to investigate the effects of antipsychotic drugs on the HPA axis in a PCP animal model of SCH.

## 1. Introduction

Schizophrenia (SCH) is a profound and enduring mental disorder with severe impacts on both individuals experiencing it and society as a whole. It is distinguished by a range of psychopathological features: positive symptoms (such as delusions and hallucinations), negative symptoms (including reduced motivation, decreased spontaneous speech, and social isolation), and cognitive deficits [1,2]. The overactivity of the mesolimbic dopaminergic pathway contributes to the emergence of positive symptoms, whereas the reduced activity of the mesocortical dopaminergic pathway is thought to play a role in manifesting negative symptoms and cognitive impairment [3]. It is believed that a combination of genetic and/or environmental factors during the development of the brain contributes to the onset of SCH [1]. 

The “two hits” hypothesis of SCH suggests that genetic susceptibility (the first hit) may predispose an individual to be more responsive to environmental insults encountered early or later in life (the second hit) [4]. The altered function of hypothalamic–pituitary–adrenal (HPA) axis activity may represent the sequel of the first hit and, therefore, can increase the overall risk for harmful effects of the second adverse event [5,6]. Increased vulnerability to environmental stress in childhood has been linked to the hyper-reactivity of the HPA and exaggerated response to acute stress in adulthood [7]. Moreover, elevated cortisol secretion may play a role in the development of psychosis [3]. Long-term exposure to hypercortisolemia has a powerful effect on dopaminergic neurotransmission [7]. The first evidence of increased dopamine release in the striatum of patients with SCH in response to psychosocial stress, measured by positron emission tomography, was provided by Mizrahi et al. [8]. However, some recent studies have shown that higher distress and anxiety were associated with lower dopamine release and cortisol response in the prefrontal cortex [9,10]. The dysregulation of HPA axis activity is consistently observed in patients with schizophrenia [11,12,13,14] and HPA activity depends mainly on the severity of the disease [7]. Elevated cortisol concentrations are found in first-episode, drug-naive SCH patients, as well as in chronically treated patients [14]. Heightened cortisol levels can impede the release of gonadotropin-releasing hormone (GnRH), luteinizing hormone (LH), follicle-stimulating hormone (FSH), and testosterone [15,16]. Alterations in sex hormones have been connected to heightened susceptibility to mood disorders in females, while testosterone has been linked to heightened sexual drive and aggression in both men and women [17]. In women, psychotic episodes tend to occur more frequently during phases of estrogen withdrawal (phase of the menstrual cycle, the post-partum period, discontinuation of estrogen therapy, and postmenopause) [18]. In men, low testosterone levels exhibit an inverse correlation with the negative symptoms of schizophrenia [19,20].

Also, patients with SCH often exhibit an impaired HPA axis response following acute stress [21,22,23]. In animal studies, male rodents show higher cortisol levels in response to stressful stimuli compared to females [24,25].

The effects of glucocorticoid hormones depend on their concentration, the number and phosphorylation of the glucocorticoid receptors, and the levels of proteins associated with the glucocorticoid receptors [26,27]. The GR is a ligand-dependent transcriptional factor. In its inactive form, GR resides in the cytoplasm bound to several heat shock proteins (HSP90 and HSP70) and co-chaperone binding (FKBP51, CyP40, p23, and Hop) proteins [27,28,29]. The phosphorylation of the GR at multiple serine/threonine residues [30] can significantly influence transcriptional activity and, consequently, cellular response to hormones [31]. Ser 211 phosphorylation is a biomarker for activated GR in vivo. The decreased expression of total GR mRNA has been found in different brain structures of SCH patients taken post-mortem [32,33,34] and in peripheral blood mononuclear cells in first-episode, unmedicated schizophrenia patients and chronic schizophrenia patients [35]. The decreased expression of GR protein was found in the brain of Wistar rats in the lipopolysaccharide-induced model of schizophrenia (LPS) [36]. FKBP51 plays a crucial role in regulating GR activity by modulating GR sensitivity to cortisol [28,37]. Increased FKBP51 expression alters GR responsiveness to cortisol, impairing the regulation of the HPA axis negative feedback loop [38]. Encoded by the FKBP5 gene, FKBP51 is strongly implicated in the pathogenesis of psychiatric disorders in certain patients [39].

11β-Hydroxysteroid dehydrogenases (11β-HSDs) are glucocorticoid-metabolizing enzymes that modulate glucocorticoid actions in a tissue-specific manner. Specifically, 11β-HSD1 converts inactive 11-keto derivatives into active glucocorticoids, thereby increasing corticosterone and cortisol tissue levels [40].

Long-term treatment with antipsychotics is frequently necessary for managing the disease effectively. “Typical” antipsychotics, such as haloperidol, predominantly block D2 receptors but they also carry a higher risk of extrapyramidal symptoms as an adverse effect. “Atypical” antipsychotics, such as clozapine, block not only D2 receptors but also serotonin 2A receptors. This dual action leads to increased dopamine release in specific brain areas, thereby decreasing motor-related side effects and potentially enhancing cognitive and emotional symptoms. These drugs have become widely used because of their greater antipsychotic efficacy and fewer extra-pyramidal side effects [41]. Unfortunately, the physical health conditions seen in individuals with SCH (such as obesity, diabetes, dyslipidemia, coronary heart disease, hypertension, and osteoporosis) are often linked to the use of antipsychotic medications, particularly atypical antipsychotics [42,43,44,45]. The dysregulation of the HPA axis presents a potential explanation for the frequent co-occurrence of compromised mental and physical well-being in individuals with SCH [46].

The administration of phencyclidine (PCP) to rodents during the perinatal period serves as a pharmacological animal model for this disorder [47,48,49,50]. This model has been widely used for many years as a valid animal model of schizophrenia [51,52,53,54]. PCP acts as a non-competitive antagonist for N-methyl-D-aspartate (NMDA) glutamatergic receptors, resulting in a wide range of outcomes in humans, encompassing both positive (agitation, audiovisual hallucinations, and paranoid delusions) and negative symptoms (such as flattening of affect and apathy), along with cognitive impairment, which closely mirrors SCH [55]. Rats subjected to perinatal PCP treatment have consistently exhibited symptoms corresponding to those seen in SCH. These symptoms include a deficit in the prepulse inhibition of acoustic stimuli, which is a specific test used to assess sensory–motor deficits in schizophrenia, an increase in locomotor activity [47,56], which is regarded as an indicator of positive symptoms, and working memory impairments, which is a fundamental aspect of cognitive dysfunction [47,53,57,58]. Disturbances in baseline temperature [59]; decreased glutathione levels and altered antioxidant defense [48,60]; a decreased number of several classes of interneurons [61]; alterations in mitochondria, apoptosis, and autophagy processes [62]; and reduced bone mass [49,63] have also been detected in rats perinatally treated with PCP. The acute administration of PCP to adult rats activates the HPA axis, causing a swift rise in the plasma levels of adrenocorticotropin and corticosterone [64].

Nonetheless, the impacts of perinatal PCP exposure on the HPA axis in adult rats have not been explored to date. To ascertain whether an increased sensitivity of the HPA axis is present, the objective of this study was to evaluate the long-lasting consequences of perinatal PCP administration and the effects of treatment with haloperidol and clozapine on corticosterone levels in the serum, along with the expression of GR, pGR, HSP70, HSP90, FKBP51, and 11β-HSD1 in several brain regions of adult male rats.

## 2. Materials and Methods

### 2.1. Animals

Twelve timed-pregnant Wistar rats were obtained on day 14 of pregnancy. The animals were housed separately in wire-hanging cages situated in a temperature-regulated animal vivarium that adhered to a 12:12 h light/dark cycle (with lights turning on at 07:00 a.m.). Food and water were accessible ad libitum throughout the experiment. Within a 12 h window following birth, the pups from the dams were cross-fostered and subsequently assigned at random to one of the nursing dams. The day of birth was designated as postnatal day 0 (PN day 0). On the 2nd, 6th, 9th, and 12th PN days, the animals were subjected to treatment, with three groups receiving PCP and three groups receiving saline (0.9% NaCl). PCP (Sigma, St. Louis, MO, USA) was dissolved in a vehicle solution of 0.9% physiological saline (0.001 g/mL) and administered subcutaneously (s.c.) in the interscapular region at a dose of 10 mg/kg. The dosage and timing of the treatment were chosen based on previously published research [47,48,49,59,60,61,62,63,65,66]. The control group received only saline, which was administered s.c. in an equivalent volume to the PCP dose. The offspring remained within their respective litters and were separated from the dams and categorized by gender upon reaching postnatal day 30, which also marked the weaning period. The present study included only male rats because of the existence of sexual dimorphism in terms of the reaction to PCP [67], and most prior investigations have been carried out using male rats [68,69,70].

### 2.2. Treatment Groups

**Figure 1:**
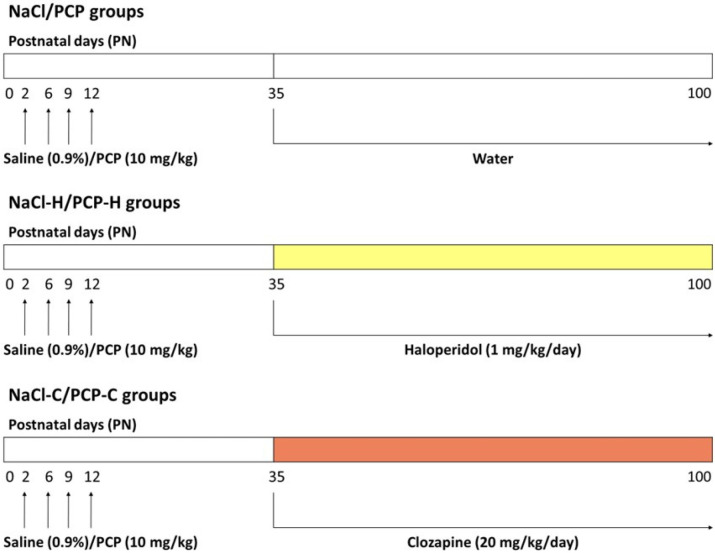
A schematic diagram of experimental design. Six groups of male animals were treated with either PCP or saline on postnatal days (PN) 2, 6, 9, and 12. (1) NaCl group (control): perinatally treated with NaCl, (2) PCP group: perinatally treated with PCP, (3) NaCl-H group: perinatally treated with NaCl and from PN day 35 received haloperidol, (4) PCP-H group: perinatally treated with PCP and from PN day 35 received haloperidol, (5) NaCl-C group: perinatally treated with NaCl and from PN day 35 received clozapine, (6) PCP-C group: perinatally treated with PCP and from PN day 35 received clozapine.

A total of six groups of male rats were examined (Figure 1):

(1) NaCl group (control): these animals received perinatal NaCl treatment (*n* = 7); starting on PN day 35, acetic acid (at a final concentration of 1mM) was introduced into the drinking water, matching the concentration used for antipsychotic dissolution.
(2) PCP group: these animals received perinatal PCP treatment (*n* = 7); starting on PN day 35, acetic acid was added to drinking water as in group 1.
(3) NaCl–H group: these animals received perinatal NaCl treatment (*n* = 7); starting on PN day 35, haloperidol (H) treatment (Krka, Slovenia) was initiated at a dosage of 1 mg/kg/day.
(4) PCP–H group: these animals received perinatal PCP treatment (*n* = 7); starting on PN day 35, this group received haloperidol therapy like group 3.
(5) NaCl–C group: these animals received perinatal NaCl treatment (*n* = 6); starting on PN day 35, clozapine (C) treatment (Sandoz, Barleben, Germany) was initiated at a dosage of 20 mg/kg/day.
(6) PCP–C group: animals received perinatal PCP treatment (*n* = 6); starting on PN35 received clozapine therapy like group 5.

The dosages of haloperidol and clozapine were determined based on molecular in vitro and in vivo occupancy studies conducted in adult animals [71,72]. The drugs were administered orally in drinking water until PN day 100. The antipsychotics were dissolved in 0.1 M acetic acid and then diluted (1:100) for daily drug administration via drinking water [73,74]. Drug administration was determined according to the average daily fluid intake and the body weight of the animals.

### 2.3. Dissection of Rats

Rats underwent a 12 h fasting period before sacrifice. Animals were sacrificed by cervical dislocation and decapitation without anesthesia on PN day 100. The heads were quickly frozen in liquid nitrogen and stored at −80 °C. Efforts were made to minimize animal distress and minimize the number of animals used in the study.

### 2.4. Determination of Corticosterone Concentration

The concentration of corticosterone was measured in serum samples using a commercially available enzyme-linked immunosorbent assay (ELISA) kit (Corticosterone EIA Kit, IDS).

Corticosterone concentration was measured in 1:10 diluted samples following the manufacturer’s instructions. Assay sensitivity was = 0.55 ng/mL. Microwell absorbance was read at 450 nm using a microplate reader. The results were calculated in comparison with the standard curve. Each sample was run in duplicate, and the average was calculated.

For the corticosterone ELISA Kit, the calculated intra-assay % CV was 4.9 for the concentration of 4.6 ng/mL and 3.8 for the concentration of 45.7 ng/mL, and the calculated inter-assay % CV was 7.8 for the concentration of 4.7 ng/mL and 7.7 for the concentration of 45.2 ng/mL.

### 2.5. Quantitative Western Blot Analysis

Animals from all experimental groups were included in Western blot analysis (n = 5 per group). The dorsolateral frontal cortex (4.2 mm up to −1.32 mm from bregma), hippocampus (−2.45 up to −3.90 mm from bregma), thalamus (−2.45 mm up to −3.70 mm from bregma), and caudate nucleus (1.8 mm up to −0.26 mm from bregma) [75] were homogenized in a lysis buffer (50 mM Tris–HCl pH 7.4, 150 mM NaCl, 1% IGEPAL CA-630, 1 mM phenylmethylsulphonyl fluoride, and protease inhibitor cocktail) on ice for 30 min, centrifuged at 18,000× *g* for 15 min at 4 °C, and the supernatants were gathered. Equivalent protein quantities from every sample were segregated using SDS-PAGE and then transferred onto nitrocellulose membranes (Bio-Rad, Hercules, CA, USA). The following primary antibodies were used in this study: GR (1:500, rabbit polyclonal, Santa Cruz, Dallas, TX, USA), pGR (Ser 211; reacts with serine phosphorylated at serine 211 in humans and serine 232 in rats) (1:1000, rabbit polyclonal, Abcam, Cambridge, UK), HSP70 (1:500, goat polyclonal, Santa Cruz, Dallas, TX, USA), HSP90 (1:5000, mouse monoclonal, Santa Cruz, Dallas, TX, USA), HSD11B1 (1:400, rabbit polyclonal, Abcam, Cambridge, UK), and FKBP51 (1:1000, rabbit polyclonal, Abcam, Cambridge, UK). Peroxidase-conjugated goat anti-rabbit, goat anti-mouse, and donkey anti-goat IgG (Southern Biotech, Birmingham, AL, USA) were used as secondary antibodies, and specific protein bands were visualized using the Chemi Doc system (Bio Rad, CA, USA). All membranes were stripped and re-probed with anti-β-Actin antibody (1:10,000, mouse monoclonal, Sigma Aldrich, St. Louis, MO, USA) to ensure that all wells were equally loaded. The protein levels were quantified by densitometry using Image Quant 5.2 software and the results were presented in relation to β-Actin.

### 2.6. Statistical Analysis

All results are presented as mean values with standard error of the mean (SEM) and were assessed using two-way ANOVA followed by Sidak’s post hoc test. IBM SPSS Statistics (Version 29.0.1.0) was used for statistical analysis. A significance level of *p* < 0.05 was considered statistically significant.

## 3. Results

### 3.1. Effects of Perinatal PCP Administration and Antipsychotic Treatment on Corticosterone Concentration in the Serum of Rats

The effects of perinatal PCP treatment, haloperidol, and clozapine on corticosterone concentration are presented in Table 1. Significant changes in corticosterone concentration [F(5,34) = 2.12, *p* < 0.05] were found between the investigated groups. Significantly decreased corticosterone concentration was detected in the PCP-H and PCP-C groups (*p* < 0.05) compared to the PCP group.

### 3.2. Effects of Perinatal PCP Administration and Antipsychotic Treatment on the Activity of GR Receptor and 11β-HSD1 Expression in the Cortex

**Figure 2:**
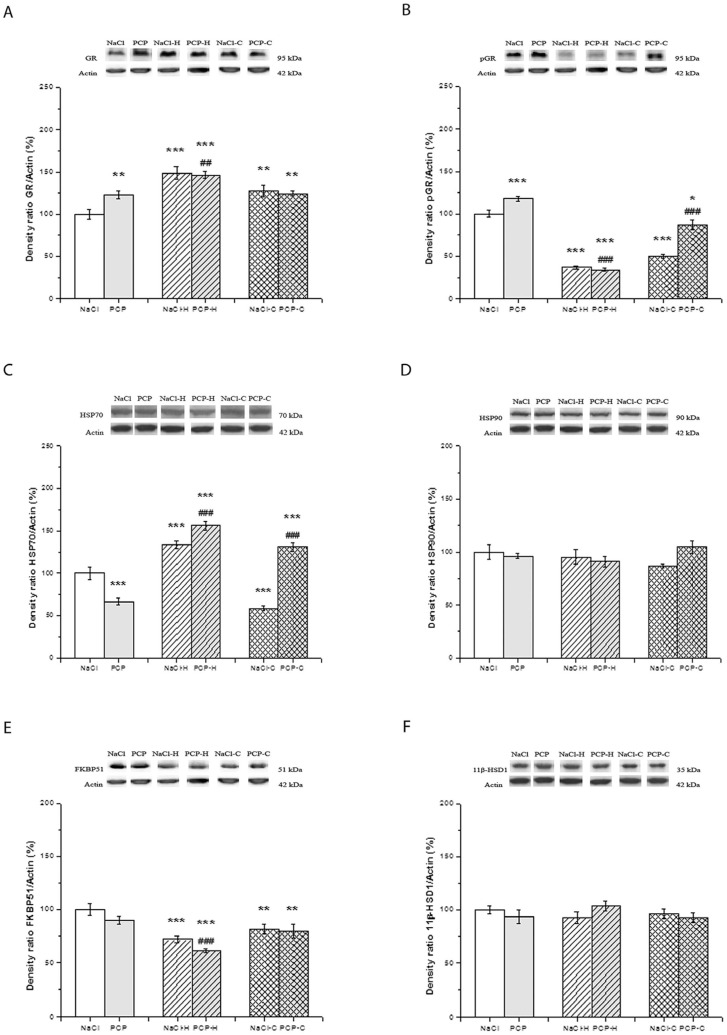
Effects of perinatal phencyclidine (PCP) treatment, haloperidol (NaCl-H and PCP-H), and clozapine (NaCl-C and PCP-C) on the expression of GR (**A**), pGR (**B**), HSP70 (**C**), HSP90 (**D**), FKBP51 (**E**), and 11β-HSD1 (**F**) in the cortex of animals. Figures are accompanied by representative Western blot bands from the same gel. Results are presented as mean values with standard error of the mean (SEM). * *p* < 0.05; ** *p* < 0.01; *** *p* < 0.001-compared to the control group; ## *p* < 0.01; ### *p* < 0.001 compared to the PCP group.

The effects of perinatal PCP administration and antipsychotics on the activity of GR, pGR, HSP70, HSP90, FKBP51, and 11β-HSD1 in the cortex are presented in Figure 2.

Two-way ANOVA revealed a significant interaction between PN treatment and drugs [F(5,24) = 3.511, *p* = 0.046] in the expression of GR (Figure 2A). Specifically, the expression was significantly elevated in the PCP (*p* = 0.007), NaCl-H (*p* < 0.001), NaCl-C (*p* = 0.005), and PCP-C groups (*p* = 0.016) compared to the control group. Furthermore, the expression of GR demonstrated a significant increase in the PCP-H group compared to both the control group (*p* < 0.001) and the PCP group (*p* = 0.017).

The expression of pGR (Figure 2B) exhibited a significant interaction between PN treatment and drugs [F(5,24) = 21.435, *p* < 0.001]. Specifically, the PCP group showed a significant increase in pGR expression (*p* < 0.001) while the NaCl-H and NaCl-C groups demonstrated a significant decrease in pGR expression compared to the control group (*p* < 0.001). Moreover, the expression of pGR was significantly reduced in the PCP-H group compared to both the control group and the PCP group (*p* < 0.001). Additionally, the PCP-C group exhibited a significant decrease in pGR expression compared to the control group (*p* = 0.048) and the PCP group (*p* < 0.001).

The expression of HSP70 (Figure 2C) demonstrated significant changes as indicated by the ANOVA [F(5,24) = 56.484, *p* < 0.001]. A significant decrease in HSP70 expression was observed in both the PCP group and NaCl-C group (*p* < 0.001), as well as a significant increase in the NaCl-H, PCP-H, and PCP-C groups (*p* < 0.001) compared to the control group. Furthermore, the expression of HSP70 was significantly elevated in both the PCP-H and PCP-C groups compared to the PCP group (*p* < 0.001).

No significant changes in the expression of HSP90 were observed between the investigated groups [F(5,24) = 2.913, *p* = 0.074] as depicted in Figure 2D.

Two-way ANOVA did not reveal a significant interaction between PN treatment and drugs concerning the expression of FKBP51 [F(5,24) = 0.738, *p* = 0.488] (Figure 2E). ANOVA showed a decrease in FKBP51 expression due to the effect of antipsychotics [F = 20.279, *p* < 0.001]. Notably, there was a significant decrease in FKBP51 expression in the NaCl-H and PCP-H groups (*p* < 0.001), as well as in the NaCl-C (*p* = 0.020) and PCP-C groups (*p* = 0.012), compared to the control group. Additionally, the expression of FKBP51 was significantly lower in the PCP-H group compared to the PCP group (*p* < 0.001).

No significant changes in the expression of 11β-HSD1 [F(5,24) = 1.704, *p* = 0.203] were observed between the investigated groups (Figure 2F).

### 3.3. Effects of Perinatal PCP Administration and Antipsychotic Treatment on the Activity of GR Receptor and 11β-HSD1 Expression in the Hippocampus

**Figure 3:**
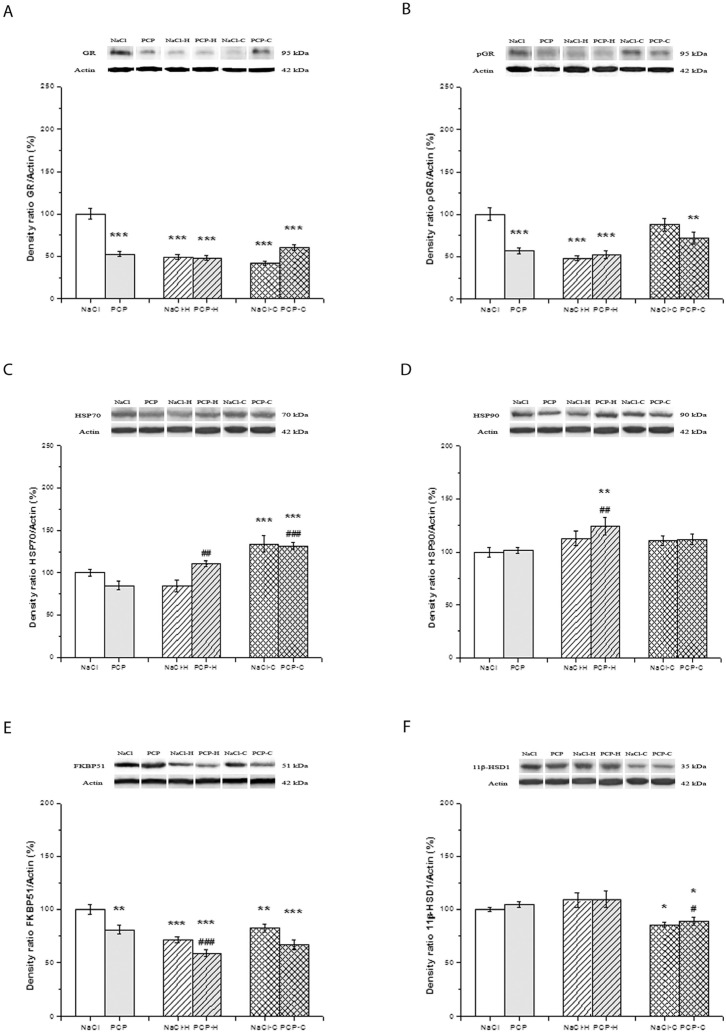
Effects of perinatal phencyclidine (PCP) treatment, haloperidol (NaCl-H and PCP-H), and clozapine (NaCl-C and PCP-C) on the expression of GR (**A**), pGR (**B**), HSP70 (**C**), HSP90 (**D**), FKBP51 (**E**), and 11β-HSD1 (**F**) in the hippocampus of animals. Figures are accompanied by representative Western blot bands from the same gel. Results are presented as mean values with standard error of the mean (SEM). * *p* < 0.05; ** *p* < 0.01; *** *p* < 0.001 compared to the control group; # *p* < 0.05; ## *p* < 0.01; ### *p* < 0.001 compared to the PCP group.

The effects of perinatal PCP administration and antipsychotics on the activity of GR, pGR, HSP70, HSP90, FKBP51, and 11β-HSD1 in the hippocampus are presented in Figure 3.

Two-way ANOVA showed a significant interaction between PN treatment and drugs [F(5,24) = 41.647, *p* < 0.001] in the expression of GR (Figure 3A). The significantly decreased expression of GR was observed in all experimental groups compared to control (*p* < 0.001).

The expression of pGR (Figure 3B) exhibited significant changes [F(5,24) = 7.818, *p* = 0.002]. A significant decrease in pGR expression was observed in the PCP, NaCl-H, PCP-H (*p* < 0.001), and PCP-C groups (*p* < 0.01) compared to the control group.

Significant changes [F(5,24) = 6.544, *p* = 0.005] were shown in the expression of HSP70 (Figure 3C). The significantly increased expression of HSP70 was seen in the NaCl-C and PCP-C groups (*p* < 0.001) compared to control. Also, the expression of HSP70 was significantly increased in the PCP-H (*p* = 0.013) and PCP-C (*p* < 0.001) groups compared to the PCP group.

Two-way ANOVA did not reveal a significant interaction between PN treatment and drugs [F(5,24) = 0.507, *p* = 0.608] in the expression of HSP90 (Figure 3D). However, the antipsychotic treatment caused significant changes [F = 4.975, *p* = 0.016]. Sidak’s post hoc test showed a significantly increased expression of HSP90 in the PCP-H group (*p* = 0.028) compared to both the control and PCP groups.

No significant interaction was observed between PN treatment and drugs [F(5,24) = 0.297, *p* = 0.746] in the expression of FKBP51 (Figure 3E). Perinatal treatment alone [F(5,24) = 23.424, *p* < 0.001], as well as drugs alone [F(5,24) = 23.424, *p* < 0.001], caused significant differences among the investigated groups. Post hoc testing showed a significant decrease in FKBP51 expression in the PCP (*p* = 0.003), NaCl-H (*p* < 0.001), PCP-H (*p* = 0.034), NaCl-C (*p* = 0.015), and PCP-C groups (*p* = 0.010) compared to the control. Additionally, the expression of FKBP51 was significantly lower in the PCP-H (*p* = 0.002) compared to the PCP group.

Two-way ANOVA showed no significant interactions between PN treatment and drugs in the expression of 11β-HSD1 [F(5,24) = 0.103, *p* = 0.902] (Figure 3F). On the other hand, drug treatment caused significant changes [F = 18.770, *p* < 0.001]. A significantly decreased expression was shown by the post hoc test in the NaCl-C (*p* = 0.018) and PCP-C groups (*p* = 0.028) compared to the control group. Also, the expression of 11β-HSD1 was significantly decreased in the PCP-C group compared to the PCP group (*p* = 0.010).

### 3.4. Effects of Perinatal PCP Administration and Antipsychotic Treatment on the Activity of GR Receptor and 11β-HSD1 Expression in the Thalamus

**Figure 4:**
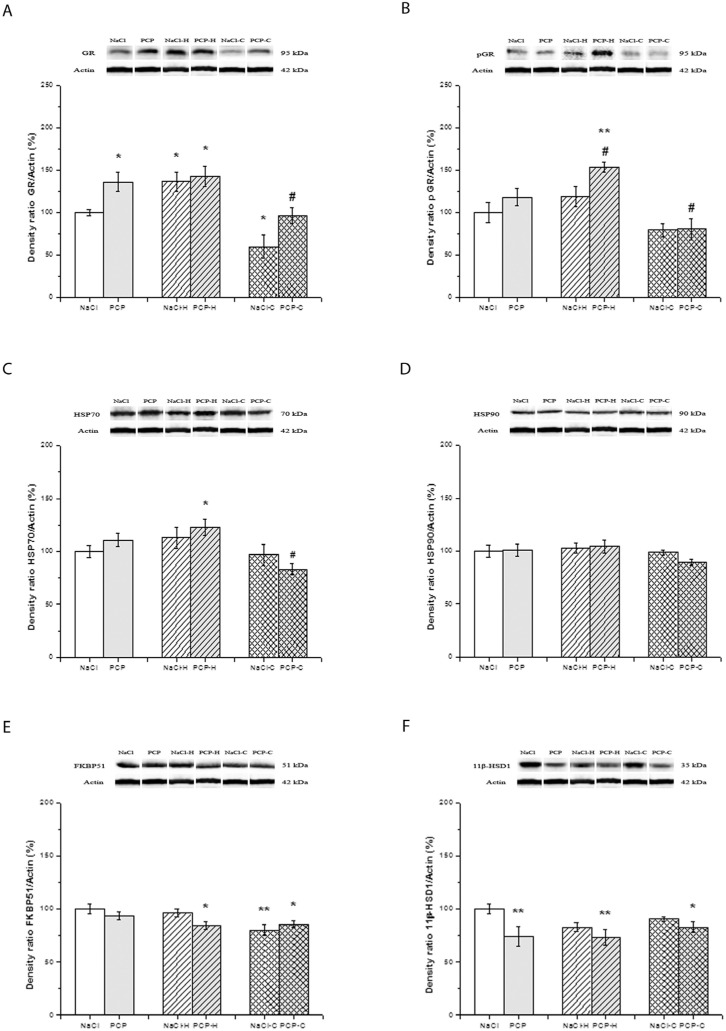
Effects of perinatal phencyclidine (PCP) treatment, haloperidol (NaCl-H and PCP-H), and clozapine (NaCl-C and PCP-C) on the expression of GR (**A**), pGR (**B**), HSP70 (**C**), HSP90 (**D**), FKBP51 (**E**), and 11β-HSD1 (**F**) in the thalamus of animals. Figures are accompanied by representative Western blot bands from the same gel. Results are presented as mean values with standard error of the mean (SEM). * *p* < 0.05; ** *p* < 0.01 compared to the control group; # *p* < 0.05 compared to the PCP group.

The effects of perinatal PCP administration and antipsychotics on the activity of GR, pGR, HSP70, HSP90, FKBP51, and 11β-HSD1 in the thalamus are presented in Figure 4.

Although significant interactions between PN treatment and drugs were not observed in the expression of GR [F(5,24) = 2.016, *p* = 0.155], drug treatment caused significant changes [F = 16.579, *p* < 0.001] (Figure 4A). A post hoc analysis revealed a significant increase in GR expression in the PCP (*p* = 0.028), NaCl-H (*p* = 0.043), and PCP-H groups (*p* = 0.041) compared to the control group. Furthermore, a significant decrease in GR expression was found in the NaCl-C group (*p* = 0.045) compared to the control group. Also, the expression of GR was significantly lower in the PCP-C group compared to the PCP group (*p* = 0.049).

No significant interactions were observed between PN treatment and drugs [F(5,24) = 1.704, *p* = 0.203] in the expression of pGR (Figure 4B). Still, perinatal treatment [F(5,24) = 5.420, *p* = 0.029] and drugs alone [F(5,24) = 16.733, *p* < 0.001] significantly changed the expression of pGR. The significantly increased expression of pGR in the PCP-H group was shown compared to the control (*p* = 0.029) and compared to the PCP group (*p* = 0.032). Also, the expression of pGR was significantly decreased in the PCP-C group compared to the PCP group (*p* < 0.043).

Two-way ANOVA showed no significant interactions between PN treatment and drugs in the expression of HSP70 [F(5,24) = 2.093, *p* = 0.145]. However, the antipsychotic treatment caused significant changes [F = 7.646, *p* = 0.003] (Figure 4C). The post hoc test showed a significant increase in the expression of HSP70 in the PCP-H group compared to the NaCl group (*p* = 0.042). Conversely, in the PCP-C group, there was a significant decrease compared to the PCP group (*p* = 0.026).

No significant changes [F(5,24) = 0.739, *p* = 0.488] were found in the expression of HSP90 between the investigated groups (Figure 4D).

The expression of FKBP51 (Figure 4E) was not significantly changed [F(5,24) = 2.492, *p* = 0.104] concerning interactions between independent variables (PN treatment and drugs). However, the treatment with antipsychotics revealed significant differences [F(5,24) = 6.286, *p* = 0.006]. A significantly decreased expression of FKBP51 was shown by post hoc testing in the PCP-H (*p* = 0.049), NaCl-C (*p* = 0.005), and PCP-C groups (*p* = 0.038) compared to the control.

The expression of 11β-HSD1 (Figure 4F) exhibited no significant changes [F(5,24) = 1.397, *p* = 0.267] concerning interactions between PN treatment and drugs. Yet, PN treatment alone caused significant differences [F(5,24) = 8.679, *p* = 0.007]. A significant decrease in the expression of 11β-HSD1 in the PCP (*p* = 0.005) and PCP-H groups (*p* = 0.008), as well as the PCP-C group (*p* = 0.022), compared to the control group, was revealed by post hoc analysis.

### 3.5. Effects of Perinatal PCP Administration and Antipsychotic Treatment on the Activity of GR Receptor and 11β-HSD1 Expression in the Caudate Nucleus

**Figure 5:**
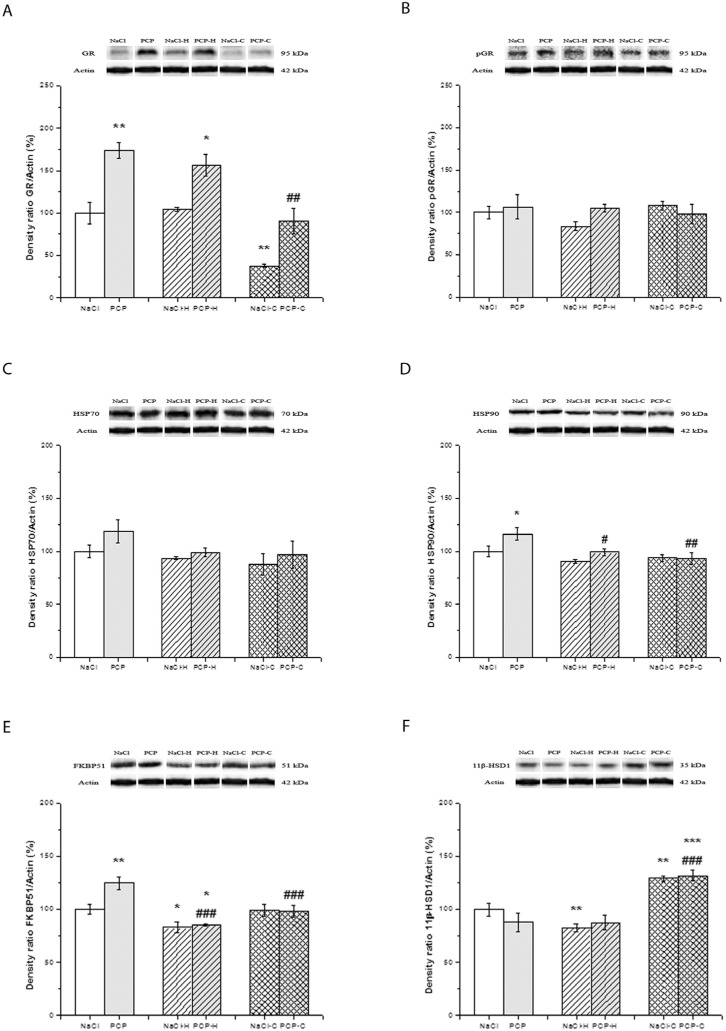
Effects of perinatal phencyclidine (PCP) treatment, haloperidol (NaCl-H and PCP-H), and clozapine (NaCl-C and PCP-C) on the expression of GR (**A**), pGR (**B**), HSP70 (**C**), HSP90 (**D**), FKBP51 (**E**), and 11β-HSD1 (**F**) in the caudate nucleus of animals. Figures are accompanied by representative Western blot bands from the same gel. Results are presented as mean values with standard error of the mean (SEM). * *p* < 0.05; ** *p* < 0.01; *** *p* < 0.001 compared to the control group; # *p* < 0.05; ## *p* < 0.01; ### *p* < 0.001 compared to the PCP group.

The effects of perinatal PCP administration and antipsychotics on the activity of GR, pGR, HSP70, HSP90, FKBP51, and 11β-HSD1 in the caudate nucleus are presented in Figure 5.

Two-way ANOVA did not reveal significant interactions between PN treatment and drugs regarding the expression of GR [F(5,24) = 0.743, *p* = 0.486] (Figure 5A). However, PN treatment alone [F(5,24) = 49.195, *p* < 0.001], as well as drugs alone [F(5,24) = 30.266, *p* < 0.001], caused significant differences among investigated groups. A significant increase in GR expression was observed by post hoc testing in the PCP (*p* < 0.001) and PCP-H group (*p* = 0.002) compared to control. Conversely, there was a significant decrease in GR expression in the NaCl-C group (*p* < 0.001) compared to the control. Also, the GR expression was significantly decreased in the PCP-C group compared to the PCP group (*p* < 0.001).

No significant changes [F(5,24) = 1.580, *p* = 0.227] were observed in the expression of pGR between investigated groups (Figure 5B).

There were no significant changes [F(5,24) = 0.341, *p* = 0.715] observed in the expression of HSP70 among the investigated groups (Figure 5C).

Two-way ANOVA did not reveal significant interactions between PN treatment and drugs regarding the expression of HSP90 [F(5,24) = 1.926, *p* = 0.168] (Figure 5D). However, PN treatment alone [F(5,24) = 4.924, *p* = 0.036], as well as drugs alone [F(5,24) = 6.924, *p* = 0.004], caused significant differences between the investigated groups. Post hoc testing showed a significantly increased expression of HSP90 in the PCP group (*p* = 0.015) compared to the control. However, the expression of HSP90 was significantly decreased in the PCP-H (*p* = 0.032) and PCP-C groups (*p* = 0.003) compared to the PCP group.

Significant changes [F(5,24) = 4.025, *p* = 0.0311] were observed in the expression of FKBP51 concerning interactions between independent variables (PN treatment and drugs) (Figure 5E). Post hoc analysis showed a significantly increased expression of FKBP51 in the PCP group (*p* = 0.001), while a significant decrease in expression was observed in the NaCl-H (*p* = 0.022) and PCP-H groups (*p* = 0.025) compared to the control group. Furthermore, the expression of FKBP51 was significantly lower in the PCP-H (*p* < 0.001) and PCP-C groups (*p* = 0.002) compared to the PCP group.

The interactions between PN treatment and drugs were not significant regarding the expression of 11β-HSD1 [F(5,24) = 1.759, *p* = 0.194] (Figure 5F). However, drugs alone caused significant differences between the investigated groups [F(5,24) = 37.366, *p* < 0.001]. Post hoc testing revealed a significant decrease in the expression of 11β-HSD1 in the NaCl-H group (*p* = 0.041) and a significant increase in the NaCl-C (*p* = 0.004) and PCP-C (*p* < 0.001) groups compared to the control group. Additionally, the expression of 11β-HSD1 was significantly higher in the PCP-C group (*p* < 0.001) compared to the PCP group.

Original membranes from Western blot analyses for each investigated antibody in specific brain structures are shown in Figure S1, while Figure 2, Figure 3, Figure 4 and Figure 5, which include each data point on the graphs, are presented in Figure S2.

## 4. Discussion

To the best of our knowledge, this is the first study in which the effects of antipsychotic drugs on the HPA axis in a PCP animal model of SCH have been investigated. This study has demonstrated the long-term effects of perinatal PCP treatment on the HPA axis, manifested in all investigated structures except the hippocampus, as the increased sensitivity of the GR signaling system. Furthermore, the results of the present study have shown that treatment with haloperidol or clozapine leads mainly to the decreased sensitivity of the GR.

### 4.1. Long-Term Effects of Perinatal PCP Treatment on the HPA Axis

The glucocorticoid receptor is widely expressed throughout the body and is also present in dopaminergic and dopaminoceptive circuits involved in psychotic symptoms [76]. Stress-induced sensitization and subsequent glucocorticoid imbalance can initiate a series of events that disrupt neural circuits, ultimately leading to dopamine system dysfunction. This process is influenced by both genetic predispositions and environmental factors [77]. In relation to the hyperdopaminergic activity observed in the mesolimbic region in schizophrenia, it is proposed that the HPA axis initiates a series of events leading to GR dysfunction, which may enhance the activity of dopamine pathways implicated in schizophrenia and related psychotic disorders [77]. The findings of our study indicate that the perinatal administration of phencyclidine does not have lasting effects on basal serum corticosterone levels. However, it significantly alters the expression of GR, pGR, and related chaperones in the brain. This suggests an increased reactivity of the GR signaling system across all examined brain regions in the experimental animals with the exception of the hippocampus, where changes point to reduced sensitivity. In accordance with our results is the study of Amani et al. [78] that examined the effects of postnatal NMDA receptor blockade on body weight, corticosterone levels, and anxiety- or depression-related behavior in adult male and female mice. The study found that the neonatal administration of PCP led to reduced body weight during both the neonatal and adult stages, without affecting baseline corticosterone levels in either male or female mice. Additionally, the research provided evidence that a 10 mg/kg dose of PCP elevated stress-induced corticosterone levels and increased anxiety- and depression-related behaviors in males while in female mice, it decreased anxiety levels without significantly impacting depression in adulthood [78]. Using a prenatal injection with polyinosinic–polycytidylic acid potassium salt as a mouse model of maternal immune activation, a reduction in the nuclear translocation of GR in the frontal cortex was noticed [79].

Our findings of HPA axis disturbances in an animal model of SCH are in agreement with the findings of the dysregulation of HPA axis activity in SCH patients [14]. However, the finding of normal corticosterone concentration in PCP perinatally treated rats in our study differs from the findings of elevated cortisol concentrations in the first-episode, drug-naive schizophrenia patients without the influence of antipsychotic medication [14]. The observed discrepancy could be related to the fact that in all studies with SCH subjects, patients were likely experiencing some degree of psychological stress due to their psychotic symptoms and/or the process of being admitted to a psychiatric hospital. Therefore, it cannot be definitively concluded that these studies demonstrate HPA axis dysfunction *per se*, as elevated cortisol levels could simply reflect a normal HPA axis response to stress. Furthermore, our findings of the increased expression of GR and/or pGR and the altered expression of related heat shock and co-chaperone proteins in the cortex, thalamus, and caudate nucleus indicate the presence of the increased sensitivity of the GR signaling system despite the normal serum corticosterone concentration in the PCP perinatally treated rats. The significance of HPA axis changes in schizophrenia is further underscored in several animal models of SCH. A translational study by Zimmerman et al. [80] analyzed the effects of varying qualities of social support and stress throughout life on glucocorticoid-responsive co-expression networks across different brain regions using a mouse model with limited nesting and bedding material from PN2 to PN9. The authors found that different qualities of social experiences significantly influenced glucocorticoid-responsive co-expression, particularly in the hippocampus and hypothalamus. Furthermore, the study of Boero et al. [81] demonstrates that social isolation in male rats alters basal HPA axis activity and impairs glucocorticoid-mediated negative feedback following acute stress.

Basta-Kaim et al. [36] in a study of lipopolysaccharide-induced schizophrenia (LPS) showed that behavioral changes accompanied by HPA axis dysregulation are characterized by elevated basal corticosterone levels in both male and female Wistar rats, which is accompanied by reduced GR expression in the hippocampus. This finding indicates that in the current LPS animal model of schizophrenia, HPA axis hyperactivity was associated exclusively with changes in the hippocampus but not with the cortical level of GR. Hippocampal glucocorticoid receptors are believed to play a role in an inhibitory feedback loop, and a reduction in these receptor levels within the hippocampus appears to suggest the hyperactivity of the HPA axis. The results of this study are partially in agreement with our research. However, in our study, the changes observed in the hippocampus suggest a reduced sensitivity of the GR signaling system, since a reduced expression of GR and pGR was demonstrated due to the perinatal administration of PCP.

Phosphorylation is a crucial mechanism for regulating the function of the glucocorticoid receptor. In humans, GR can be phosphorylated at serine 211 (pGR-S211) and serine 226 (pGR-S226) by various kinases, including cyclin-dependent kinases and mitogen-activated protein kinases [26,82,83]. The GR phosphorylation at S211 (corresponding to S232 in rats) was shown to promote hormone-dependent GR translocation to the nucleus and to significantly enhance its transcriptional activity [30,84,85,86]. On the other hand, the phosphorylation of GR at S226 inhibits its transcriptional activity and promotes its nuclear export after hormone withdrawal [30,85,87]. We have found structure-specific changes in the GR and pGR-S232 in the PCP perinatally treated rats. In the cortex, both GR and pGR were elevated; in the thalamus and caudate nucleus, only GR was elevated; meanwhile, in the hippocampus, both GR and pGR were decreased.

Heat shock proteins, which are essential for the folding and regulation of a wide range of cellular proteins, are increasingly recognized as critical players in various brain processes, including neurite outgrowth [88], neuronal differentiation [89], polarization [90], and neurodegeneration [91]. HSP70 facilitates the folding of GR into a low-steroid-affinity conformation while HSP90 is an important component of a complex network of molecular chaperones (a heterocomplex) that facilitates the conformational changes required for the GR and other steroid receptors to translocate into the nucleus. Once in the nucleus, these receptors can carry out their genomic functions as part of the mature complex. [92]. In our study, the only change in HSP70 in PCP perinatally treated rats was in the cortex, where its decreased expression was observed. This is opposite to the acute effect of PCP or the other NMDA receptor antagonist dizocilpine (MK-801), which are shown to increase the expression of HSP70 [93,94]. However, the decreased expression of HSP70 in our study indicates the increased affinity of the receptor for the hormone, and together with the finding of the increased expression of GR and pGR in this structure, this suggests the increased sensitivity of the GR system. The expression of the other measured chaperone protein, HSP90, was, however, mainly unchanged in the PCP perinatally treated rats compared to control animals. This is mainly opposed to the results in SCH patients, where the increased expression of Hsp70 mRNA [95] and HSP70 protein [96] was observed in the prefrontal cortex. Considering the role of chaperones as necessary molecules for the repair and removal of cellular proteins damaged in stress, defects in their function caused by immune insults in the period of fetal growth may be involved in the neurodevelopmental mechanism of schizophrenia [97].

Immunophilin FKBP51 plays a pivotal role in the regulation of GR activity. High levels of FKBP51 are linked to GR resistance and reduced stress-coping behavior. FKBP51 is encoded by the gene FKBP5 and determines GR binding affinity to glucocorticoids. HSP90, p23, and FKBP51 stabilize the GR complex in a high-affinity state. The translocation of the cortisol-bound GR into the nucleus is facilitated when FKBP52 displaces FKBP51. Consequently, the HSP90/FKBP51 complex acts as a short-term, negative feedback regulator of GR signaling by reducing the receptor’s ligand binding affinity [28]. Variations in the function of the HSP90/FKBP51 complex can arise due to changes in FKBP5 levels, which are influenced by genetic, epigenetic, and environmental factors. FKBP5 allelic variations have been associated with schizophrenia [98]. Elevated FKBP5 expression has been observed in the prefrontal cortex [99] and cerebellum [100] of SCH patients. A recent study by Debs et al. [101] suggested that the overexpression of FKBP5 mRNA is associated with impaired negative feedback in the stress hormone response in the midbrain of SCH patients, particularly in those with high levels of neuroinflammation. In our study, the long-term effects of perinatal PCP treatment on the expression of FKBP51 were structurally specific. In perinatally PCP-treated rats, the expression of this protein remained unchanged in the cortex and thalamus while it decreased in the hippocampus and increased in the caudate nucleus. Also, our results showed that the expression of 11β-HSDs, enzymes that are responsible for metabolizing glucocorticoids and regulating the intracellular levels of steroids available to activate corticosteroid receptors, was mainly unchanged in the brains of animals treated perinatally with PCP.

Taken together, our results in rats perinatally treated with PCP that correspond to the drug-naïve patients indicate structure-specific changes in GR signaling. The most prominent results are the increased sensitivity of GR signaling in the cortex and decreased sensitivity in the hippocampus. It seems that perinatal PCP administration leads to the activation of GRs in the cortex, thalamus, and caudate nucleus, causing the increased expression of GR and pGR and different changes in the investigated heat shock proteins. Interestingly, the expression of FKBP51 in our study was increased in the caudate nucleus, speaking in favor of the impaired sensitivity of the GR. Our findings of more sensitive GRs in an animal model of psychosis suggest that the changes in GR signaling could be the biological factor responsible for increased vulnerability to stress. It would be interesting to investigate the influence of stress on these parameters in the brain of PCP perinatally treated rats.

### 4.2. Effects of Antipsychotic Treatment on the HPA Axis

Long-term treatment with either “typical” or “atypical” antipsychotics is often necessary to manage schizophrenia symptoms. Over the past few decades, numerous studies have reported that antipsychotic medication influences basal HPA axis activity [11,14,102]. Both first- and second-generation antipsychotics have been found to reduce plasma cortisol concentrations in schizophrenia patients, with more studies indicating that second-generation antipsychotics decrease cortisol levels more significantly than first-generation ones [103,104,105]. In our study, we observed similar effects of antipsychotics on corticosterone levels in perinatally PCP-treated rats. Haloperidol or clozapine administration in these groups resulted in a significant decrease in corticosterone compared to PCP-treated rats without antipsychotics. Additionally, antipsychotics alone did not affect corticosterone concentrations, a finding consistent with Samadi et al. [106]’s study that demonstrated that the intraperitoneal treatment of rats with haloperidol (0.5 mg/kg) or clozapine (0.5 mg/kg) for 28 days does not alter serum corticosterone levels. Treatments with haloperidol or clozapine were followed by similar structure-specific changes in examined brain regions. In the cortex, haloperidol increased the expression of GR and HSP70 in both NaCl and PCP perinatally treated rats but at the same time decreased the expression of pGR and FKBP51 in both NaCl and PCP perinatally treated rats. In the cortex, clozapine also increased the expression of GR in both groups but the increased expression of HSP70 was seen only in the PCP perinatally treated group. The changes in the expression of pGR and FKBP51 in both NaCl and PCP perinatally treated rats were the same as in animals treated with haloperidol. Compared to haloperidol-treated groups, differences were seen in the higher influence on the expression of HSP70 and the absence of the changes in HSP90 in the hippocampus; the decreased expressions of the GR, pGR, and HSP70 in the thalamus; and the decreased expression of the GR in the caudate nucleus.

It seems that antipsychotic treatment results in a decrease in receptor activation, considering that it leads to a reduction in the active form of the receptor. Also, the increase in HSP70 reduces the affinity of the receptor for hormone binding. Studies have also shown that PCP significantly increases HSP70 mRNA expression in the prefrontal cortex of rats and that haloperidol potentiates—while atypical antipsychotics prevent—this increase [107]. A previous study conducted by Roh et al. [108] revealed significantly reduced HSP70 expression induced by MK-801 in rat C6 glioma cells in proportion to the haloperidol pretreatments that were extended. The results of this study are not in agreement with our results and are probably due to the different actions of individual CNS cells.

One of the most persistent findings in our research was the decreased expression of FKBP51 in the brain of rats caused by treatment with haloperidol and clozapine. Sinclair et al. [99] showed, for the first time, changes in the regulation of FKBP5 transcription in the brain of patients with schizophrenia. Namely, by post-mortem analysis, they identified an increased expression of FKBP5 mRNA in the prefrontal cortex of patients with SCH compared to the control group. Basta-Kaim et al. [36] found a decreased expression of the hippocampal GR and FKBP51 in the frontal cortex in the lipopolysaccharide (LPS)-induced neurodevelopmental model of SCH. The increase in FKBP51 expression shown in the study by Basta-Kaim et al. [36] after clozapine administration is not consistent with our results, since clozapine treatment led to a decrease in FKBP51 expression in the examined rat brain structures. The observed differences may be the result of different protocols as well as the administered dose of antipsychotics. Daskalakis and Binder [109] postulated the involvement of the FKBP5 gene within the vulnerability–stress model of SCH, particularly concerning the interplay between genes and stress. The authors suggest that, in essence, FKBP5 could represent a broader stress-modulating element that contributes to both increased susceptibility and resilience across various mental disorders by mitigating the detrimental impacts of adverse life events. In the first study that analyzed the FKBP5 haplotype in patients with schizophrenia and their healthy siblings compared to controls [98], the existence of the risk alleles and “risk” haplotype combinations in schizophrenia was demonstrated, especially when childhood trauma is present as a contributing factor.

Animal studies involving FKBP51 gene overexpression or knockout mice have been conducted to elucidate potential mechanisms for FKBP51-mediated alterations in HPA axis activity. Hartmann et al. [110] observed significant impacts on HPA axis function in their research and showed that FKBP5-knockout (51KO) mice exhibited significantly lower basal corticosterone levels compared to wild-type (WT) animals under chronic stress conditions. Their neuroendocrine data suggest that 51KO mice have enhanced negative feedback within the HPA axis, leading to reduced stress vulnerability in this regard, likely due to increased GR sensitivity. Additionally, Hoeijmakers et al. [111] examined neuroendocrine, behavioral, and physiological changes related to mood disorders in female 51KO mice. In line with this, mice treated with a highly specific FKBP51 antagonist also exhibited decreased basal corticosterone secretion [112]. This finding further supports the hypothesis that FKBP51 plays a role in regulating HPA axis activity [38].

The significance of our findings is supported by the new evidence suggesting that exposure to stressful events early in life can modify the epigenomic landscape [113]. Epigenetic modifications (i.e., DNA methylation, histone modifications, and noncoding RNAs) can effectively alter the genome’s transcription without changes in nucleotide sequence [109]. Strong evidence indicates that epigenetic alterations in genes associated with stress and the glucocorticoid signaling pathway serve as a mechanism by which the effects of stress-related experiences become imprinted within an individual’s biology [114].

We must acknowledge several limitations in our study. First, we did not assess adrenal gland weight, nor did we measure plasma corticotrophin-releasing hormone (CRH) and adrenocorticotropic hormone (ACTH) levels, which, in turn, stimulate the adrenal cortex to produce corticosteroids. Second, we did not conduct dose–response studies with antipsychotics, and, therefore, we can only speculate that the disparities observed in brain structures are linked to advanced alterations in HPA axis activity concerning the duration of antipsychotic treatment. Third, we administered antipsychotics orally, which allowed for variations in drug intake by the animals. Even though this approach can mimic the situation with SCH patients who frequently exhibit irregular medication adherence, further investigations involving longer durations, varying doses, and diverse routes of antipsychotic administration are required. Fourth, we used a limited sample size, and, lastly, since our measurements were confined to protein expression via immunoblots, additional experiments focused on mRNA expression, the immunohistochemical characterization of changes, and an exploration of epigenetic modifications within the genes for the GR receptor and GR-associated proteins are warranted.

## 5. Conclusions

Our findings indicate the presence of HPA axis disruptions in the PCP animal model of SCH. The perinatal administration of PCP leads to complex alterations in the glucocorticoid signaling system, indicating the heightened sensitivity of GR. Antipsychotics predominantly act protectively, reducing GR sensitivity irrespective of perinatal treatment and the specific drug used. Examining changes in GR and GR-associated proteins in the cytoplasm could enhance our understanding of the role of glucocorticoid hormones in SCH. This insight may contribute to the identification of more effective therapy. FKBP51 antagonists are currently under intensive study as potential pharmacological tools for addressing conditions marked by HPA axis hyperactivity. Exploring the involvement of other proteins influencing GR sensitivity could reveal new targets for directing therapeutic interventions. Additional research is necessary to understand the significance of HPA axis disturbances in this animal model and their potential relevance to SCH. Future experiments should include an evaluation of the immediate effects of perinatal PCP treatment, as well as an examination of the consequences of acute stressful events in adult rats subjected to perinatal PCP treatment. This comprehensive approach should include, among other things, the measurement of both coding and noncoding RNAs, the application of immunohistochemical techniques, and the investigation of epigenetic modifications within the genes for the GR and GR-associated proteins.

## Figures and Tables

**Table 1:** Effects of perinatal phencyclidine (PCP) treatment, haloperidol (H), and clozapine (C) on corticosterone concentration in the serum of adult male rats. Results are presented as mean values with standard error of the mean (SEM).

	NaCl	PCP	NaCl-H	PCP-H	NaCl-C	PCP-C
Corticosterone (ng/mL)	51 ± 8.5	67.8 ± 11.3	55.6 ± 10.4	37.6 ± 2.8 ^#^	61.7 ± 5.6	39.9 ± 3.4 ^#^

^#^ *p* < 0.05 compared to the PCP group

## Data Availability

The data that support the findings of this study are available upon request from the corresponding author.

## Supplementary Materials

The following supporting information can be downloaded at https://www.mdpi.com/article/10.3390/cells13171425/s1: Figure S1: Original membranes of Western blot analyses for each investigated antibody in specific brain structures; Figure S2: Figure 2, Figure 3, Figure 4 and Figure 5 containing each data point on the graphs.
