# Supplementary material for: Effects of Antipsychotics on the Hypothalamus–Pituitary–Adrenal Axis in a Phencyclidine Animal Model of Schizophrenia
Source: Cells. 2024 Aug 26;13(17):1425. doi: 10.3390/cells13171425 (PMC11394463; doi:10.3390/cells13171425)
Supplement: Supplementary file 1 [file cells-13-01425-s001.zip › Figure S2.pdf]

Cortex

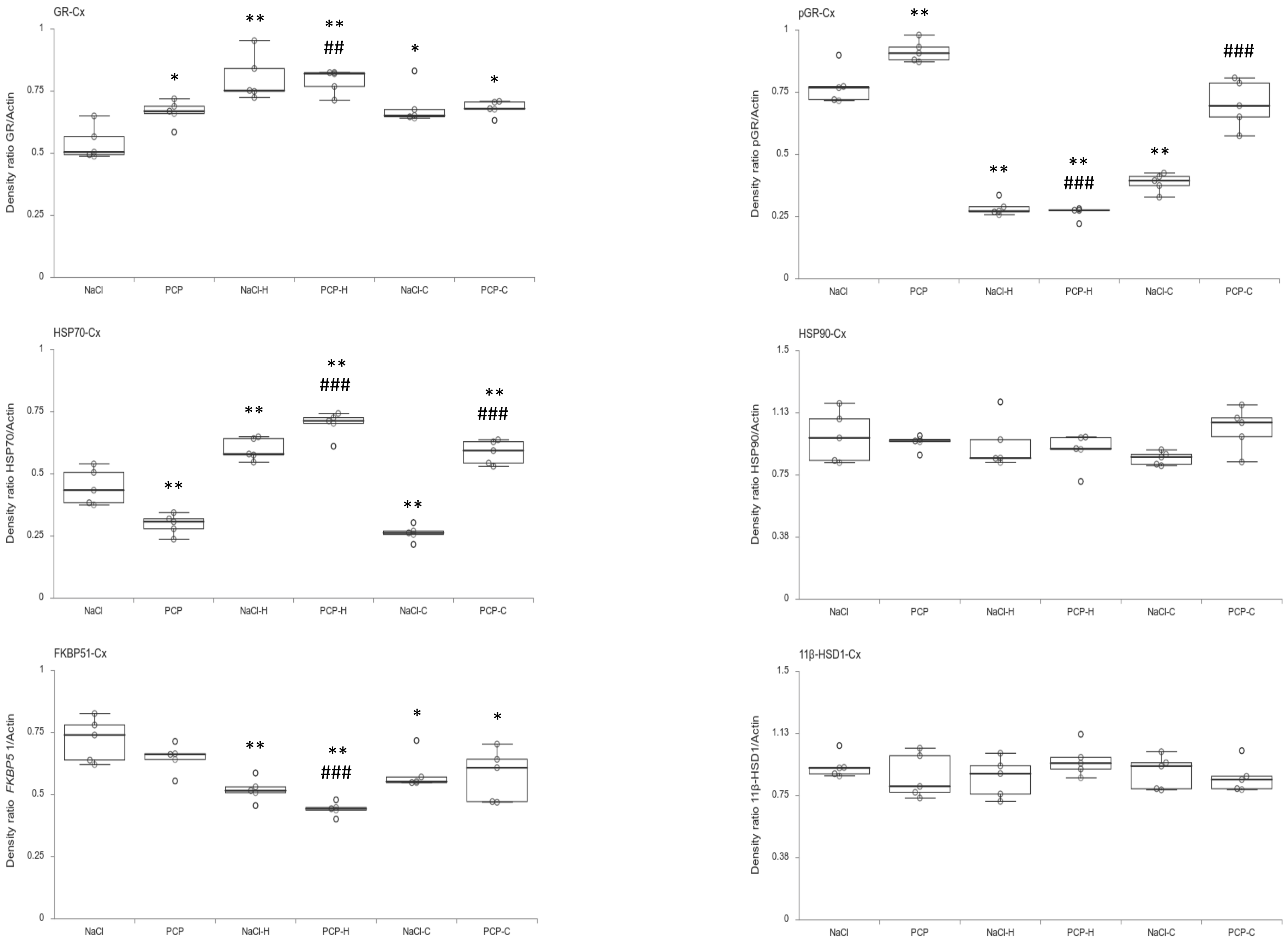

GR-Cx
Density ratio GR/Actin
pGR-Cx
Density ratio pGR/Actin
HSP70-Cx
Density ratio HSP70/Actin
HSP90-Cx
Density ratio HSP90/Actin
FKBP51-Cx
Density ratio FKBP5 1/Actin
11β-HSD1-Cx
Density ratio 11β-HSD1/Actin
NaCl
PCP
NaCl-H
PCP-H
NaCl-C
PCP-C

Hippocampus

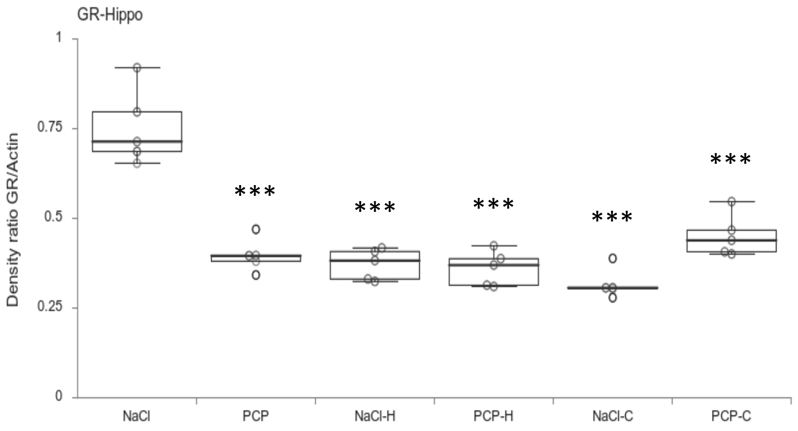

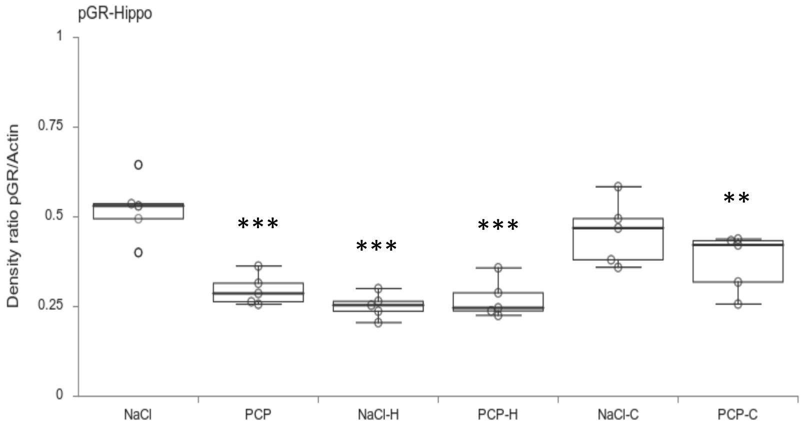

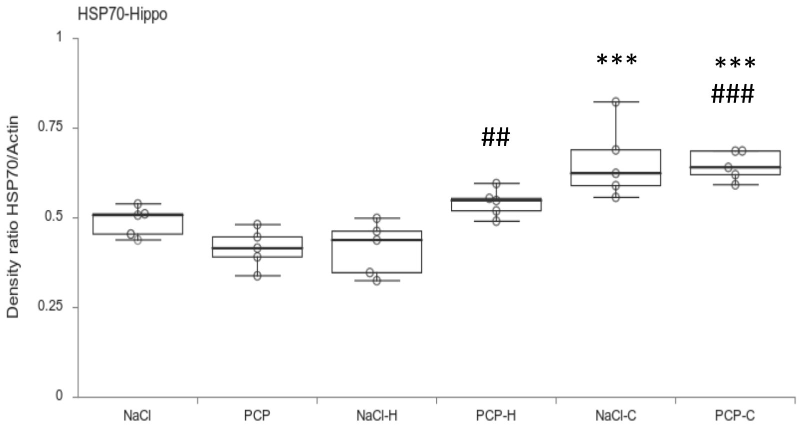

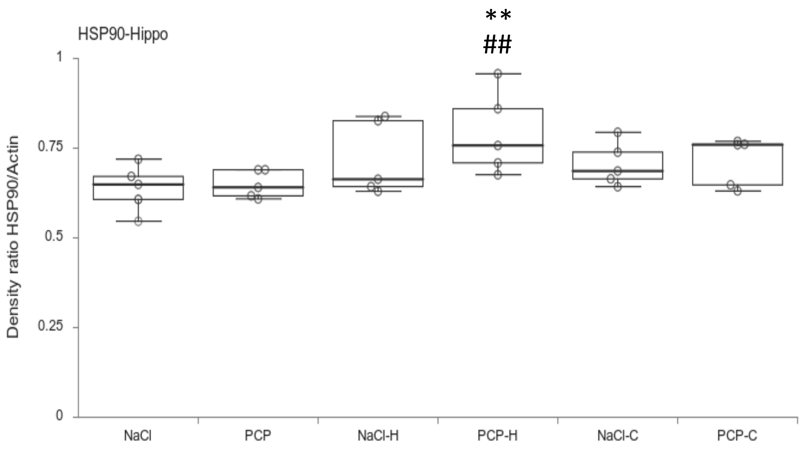

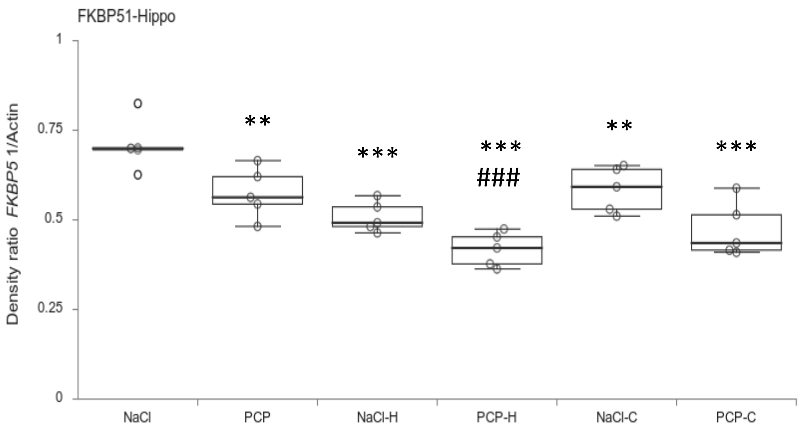

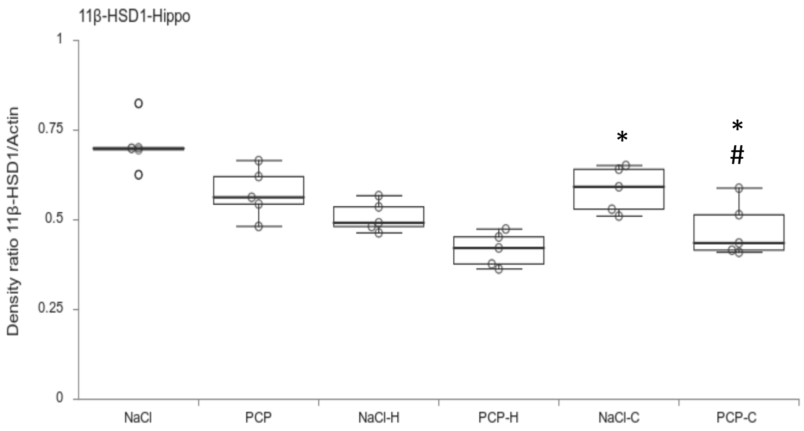

Thalamus

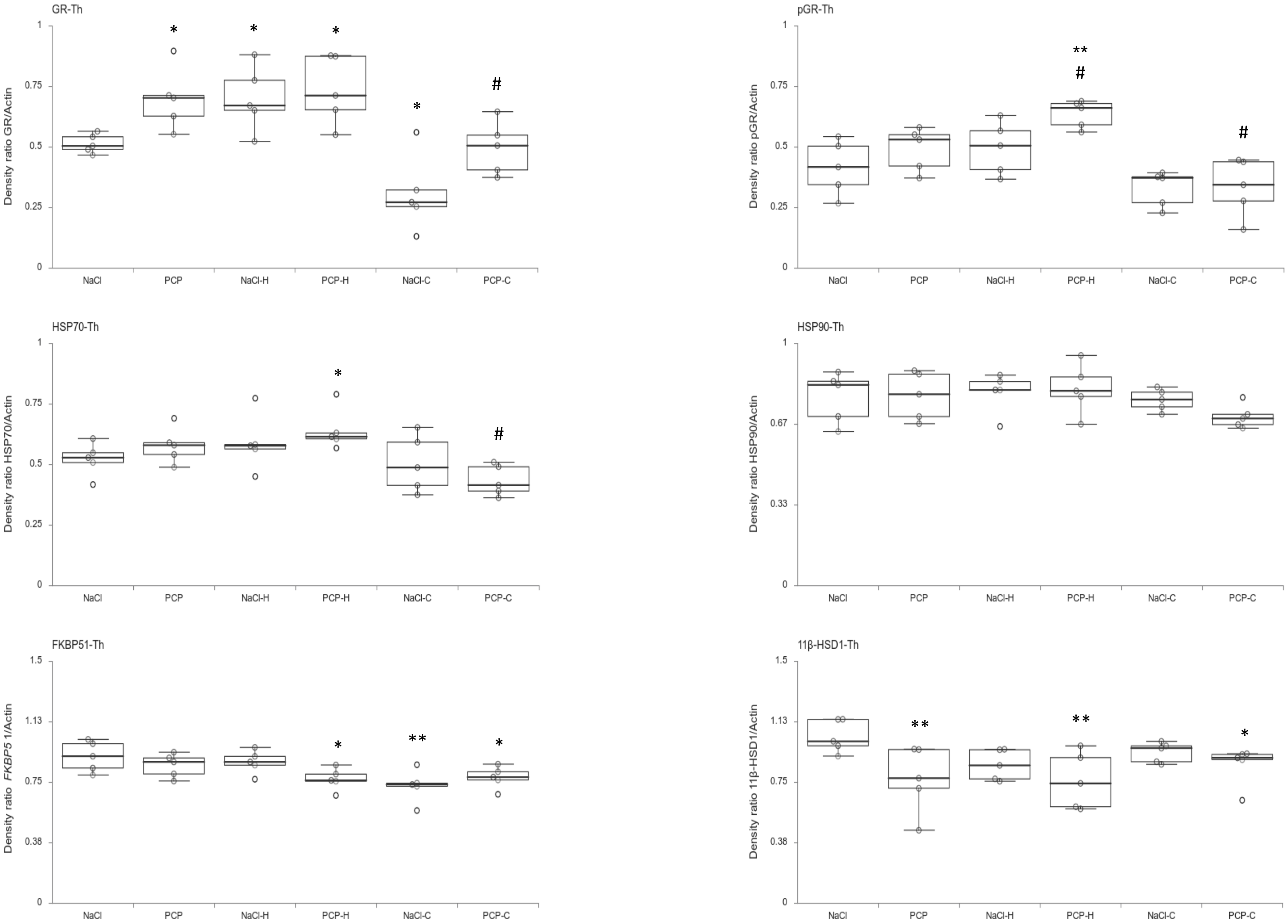
GR-Th
Density ratio GR/Actin
0
0.25
0.5
0.75
1
NaCl
PCP
NaCl-H
PCP-H
NaCl-C
PCP-C
*
*
*
*
#
pGR-Th
Density ratio pGR/Actin
**
#
#
HSP70-Th
Density ratio HSP70/Actin
*
#
HSP90-Th
Density ratio HSP90/Actin
0.33
0.67
FKBP51-Th
Density ratio *FKBP5* 1/Actin
0.38
0.75
1.13
1.5
*
**
*
11β-HSD1-Th
Density ratio 11β-HSD1/Actin
**
**
*

Caudate nucleus

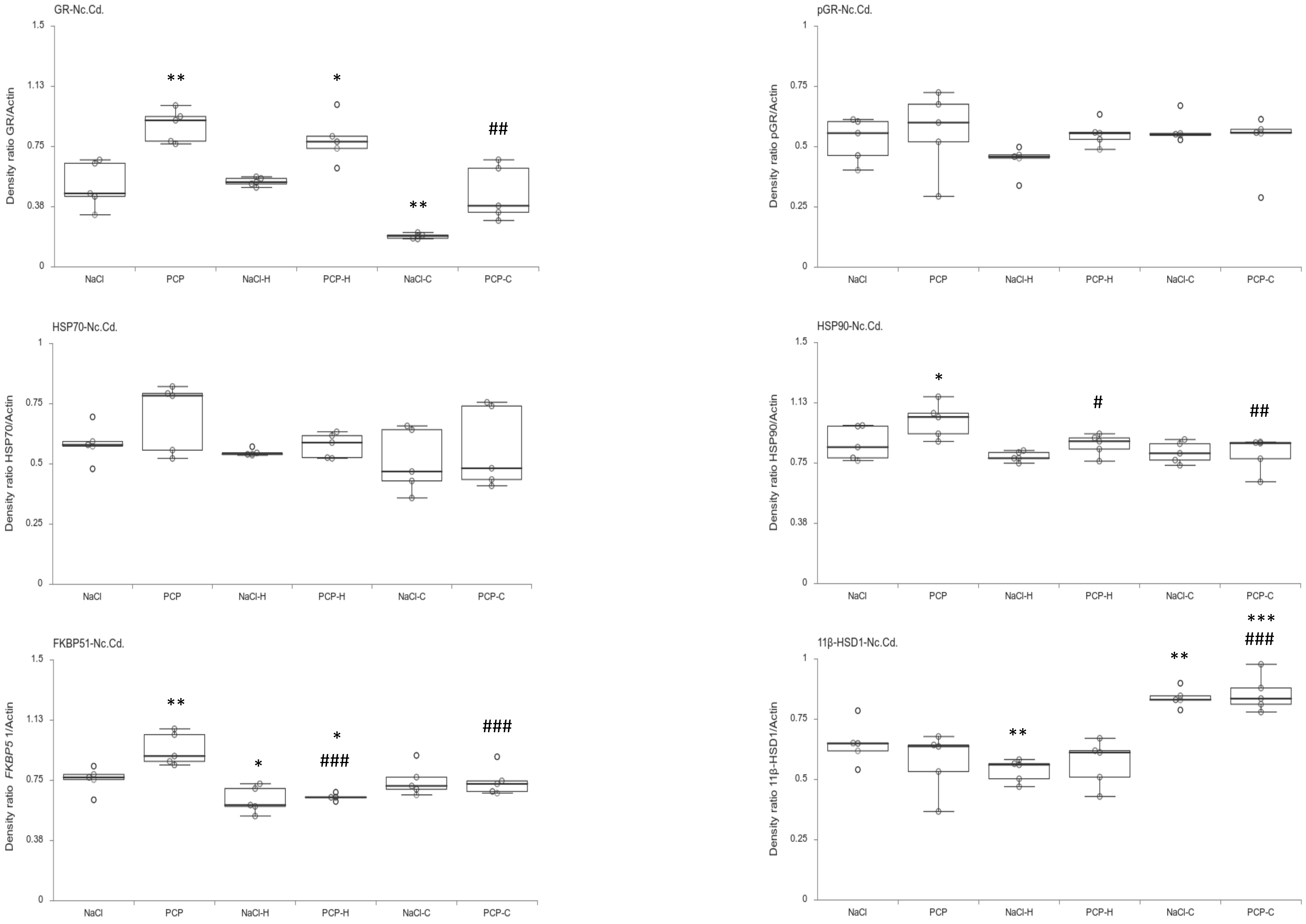

Effects of perinatal phencyclidine (PCP) treatment, haloperidol (NaCl-H and PCP-H), and clozapine (NaCl-C and PCP-C) on the expression of GR, pGR, HSP70, HSP90, FKBP51 and HSD11B1 in the cortex, hippocampus, thalamus and caudate nucleus of animals. Results are presented as box plot with data points.

*$p<0.05$; **$p<0.01$; ***$p<0.001$ - compared to the control group

#$p<0.05$; ##$p<0.01$; ###$p<0.001$ - compared to the PCP group
