# Supplementary material for: Effects of Antipsychotics on the Hypothalamus–Pituitary–Adrenal Axis in a Phencyclidine Animal Model of Schizophrenia
Source: Cells. 2024 Aug 26;13(17):1425. doi: 10.3390/cells13171425 (PMC11394463; doi:10.3390/cells13171425)
Supplement: Supplementary file 1 [file cells-13-01425-s001.zip › Figure S1.PDF]

GR

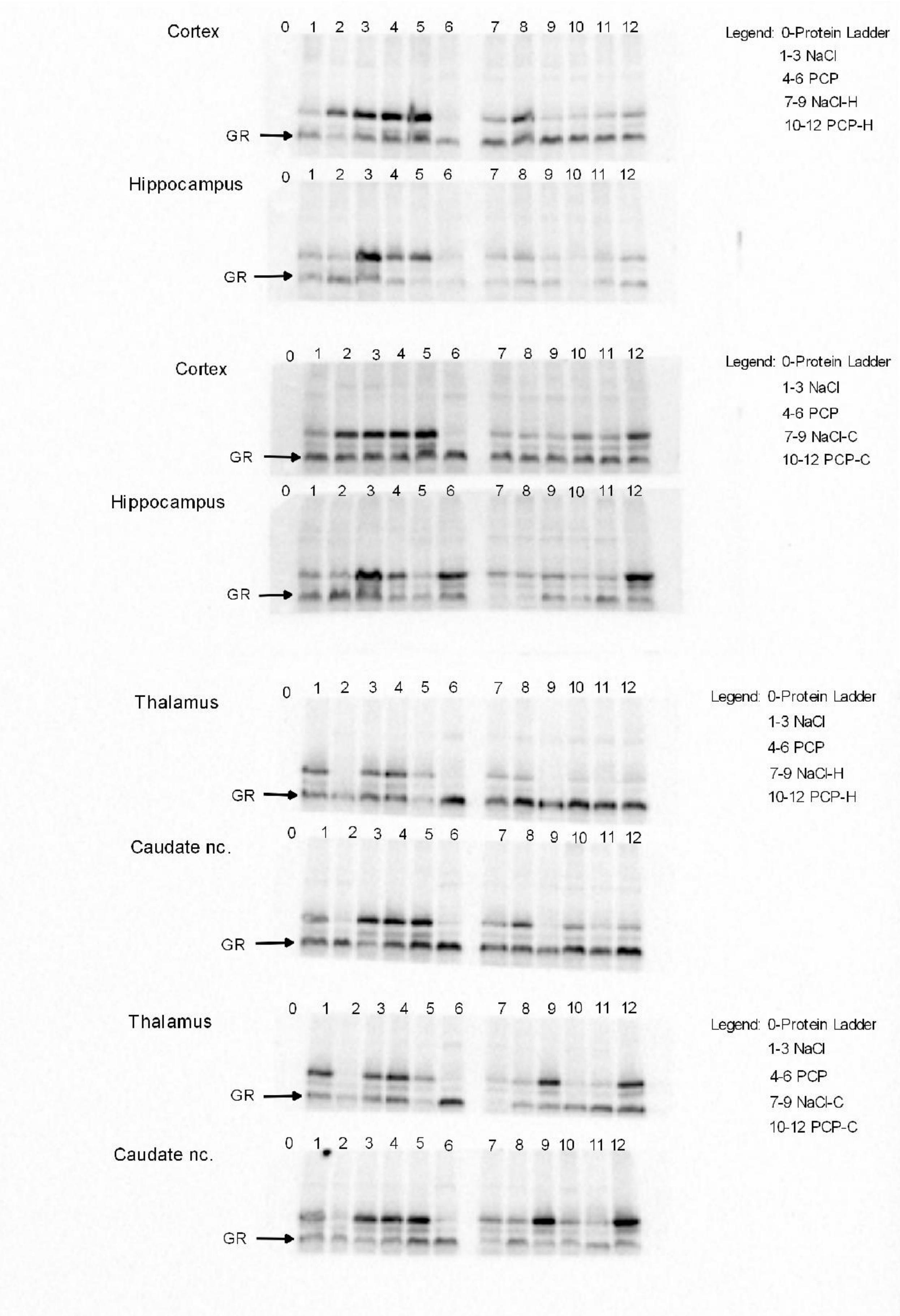
Cortex
0 1 2 3 4 5 6 7 8 9 10 11 12
GR
Legend: 0-Protein Ladder
1-3 NaCl
4-6 PCP
7-9 NaCl-H
10-12 PCP-H
Hippocampus
0 1 2 3 4 5 6 7 8 9 10 11 12
GR
Cortex
0 1 2 3 4 5 6 7 8 9 10 11 12
GR
Legend: 0-Protein Ladder
1-3 NaCl
4-6 PCP
7-9 NaCl-C
10-12 PCP-C
Hippocampus
0 1 2 3 4 5 6 7 8 9 10 11 12
GR
Thalamus
0 1 2 3 4 5 6 7 8 9 10 11 12
GR
Legend: 0-Protein Ladder
1-3 NaCl
4-6 PCP
7-9 NaCl-H
10-12 PCP-H
Caudate nc.
0 1 2 3 4 5 6 7 8 9 10 11 12
GR
Thalamus
0 1 2 3 4 5 6 7 8 9 10 11 12
GR
Legend: 0-Protein Ladder
1-3 NaCl
4-6 PCP
7-9 NaCl-C
10-12 PCP-C
Caudate nc.
0 1 2 3 4 5 6 7 8 9 10 11 12
GR

pGR

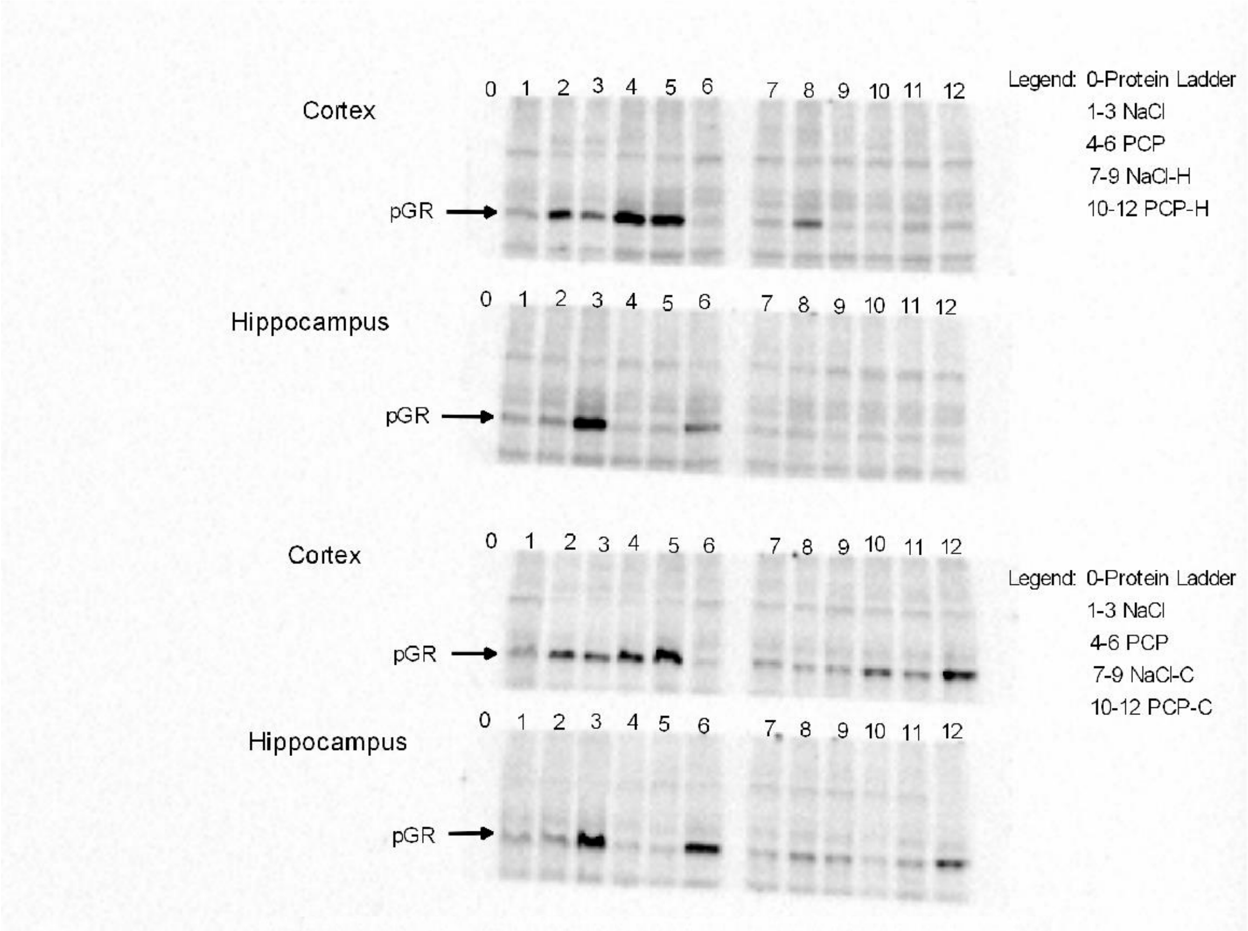
Cortex
0 1 2 3 4 5 6 7 8 9 10 11 12
pGR
Legend: 0-Protein Ladder
1-3 NaCl
4-6 PCP
7-9 NaCl-H
10-12 PCP-H
Hippocampus
0 1 2 3 4 5 6 7 8 9 10 11 12
pGR
Cortex
0 1 2 3 4 5 6 7 8 9 10 11 12
pGR
Legend: 0-Protein Ladder
1-3 NaCl
4-6 PCP
7-9 NaCl-C
10-12 PCP-C
Hippocampus
0 1 2 3 4 5 6 7 8 9 10 11 12
pGR

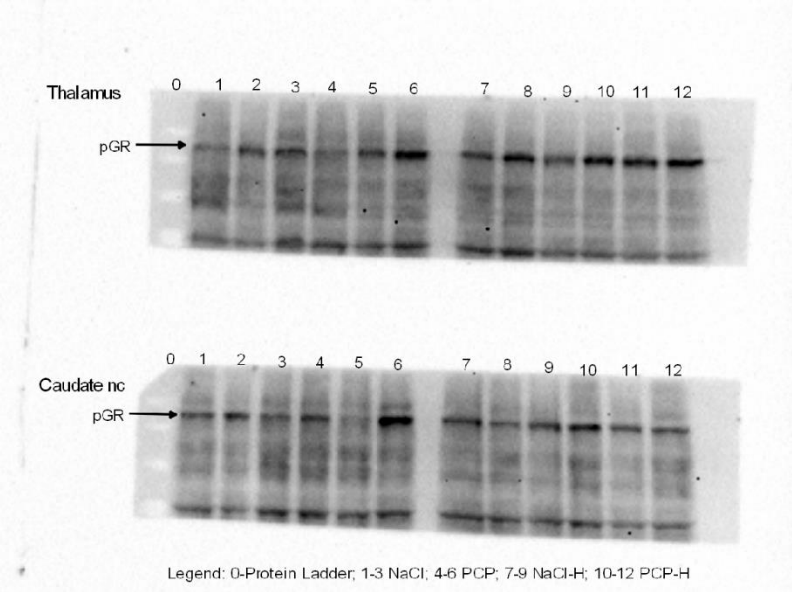
Thalamus
0 1 2 3 4 5 6 7 8 9 10 11 12
pGR
Caudate nc
0 1 2 3 4 5 6 7 8 9 10 11 12
pGR
Legend: 0-Protein Ladder; 1-3 NaCl; 4-6 PCP; 7-9 NaCl-H; 10-12 PCP-H

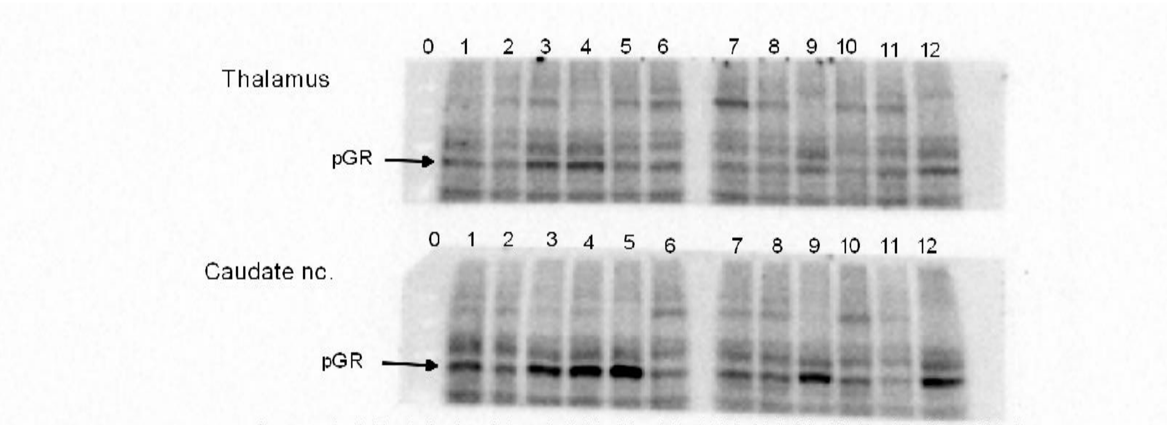

Legend: 0-Protein Ladder; 1-3 NaCl; 4-6 PCP; 7-9 NaCl-C; 10-12 PCP-C

HSP70

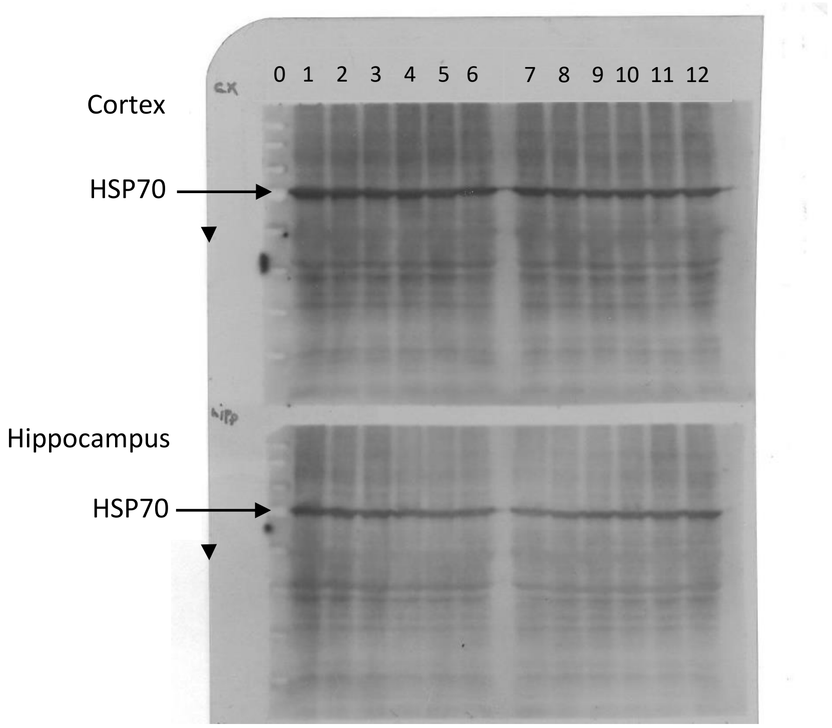

Legend: 0-Protein Ladder; 1-3 NaCl; 4-6 PCP; 7-9 NaCl-H; 10-12 PCP-H

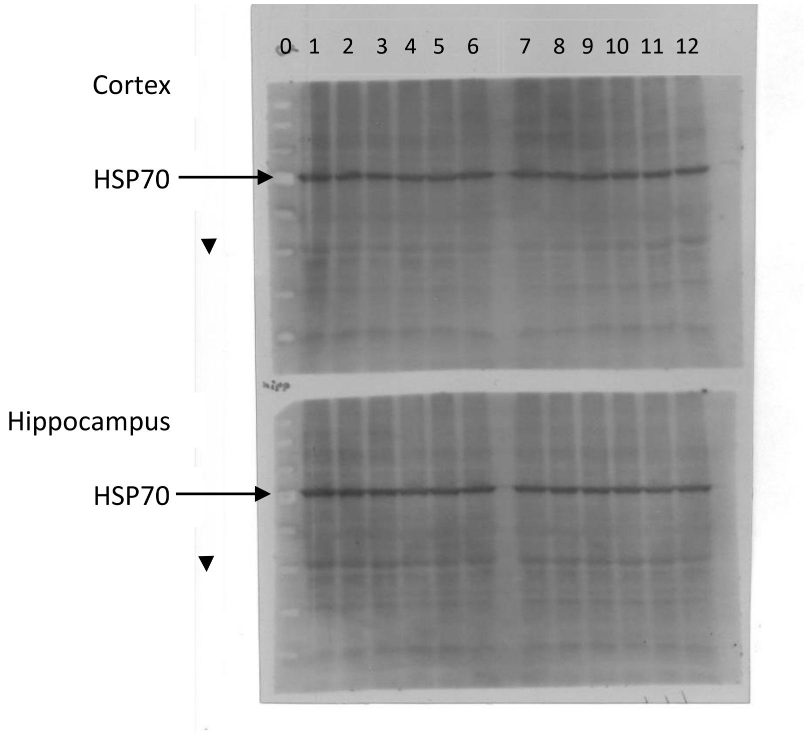

Legend: 0-Protein Ladder; 1-3 NaCl; 4-6 PCP; 7-9 NaCl-C; 10-12 PCP-C

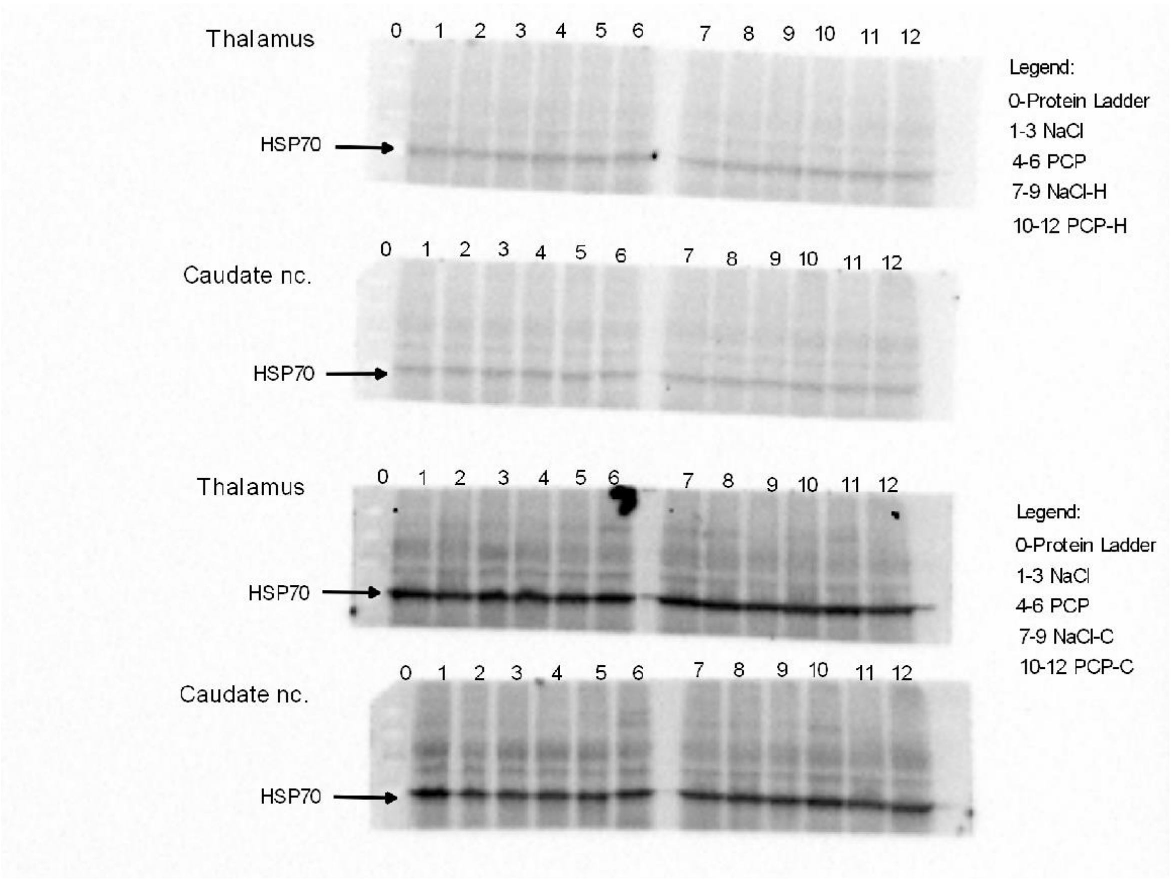
Thalamus
0 1 2 3 4 5 6 7 8 9 10 11 12
HSP70
Legend:
0-Protein Ladder
1-3 NaCl
4-6 PCP
7-9 NaCl-H
10-12 PCP-H
Caudate nc.
0 1 2 3 4 5 6 7 8 9 10 11 12
HSP70
Thalamus
0 1 2 3 4 5 6 7 8 9 10 11 12
HSP70
Legend:
0-Protein Ladder
1-3 NaCl
4-6 PCP
7-9 NaCl-C
10-12 PCP-C
Caudate nc.
0 1 2 3 4 5 6 7 8 9 10 11 12
HSP70

HSP90

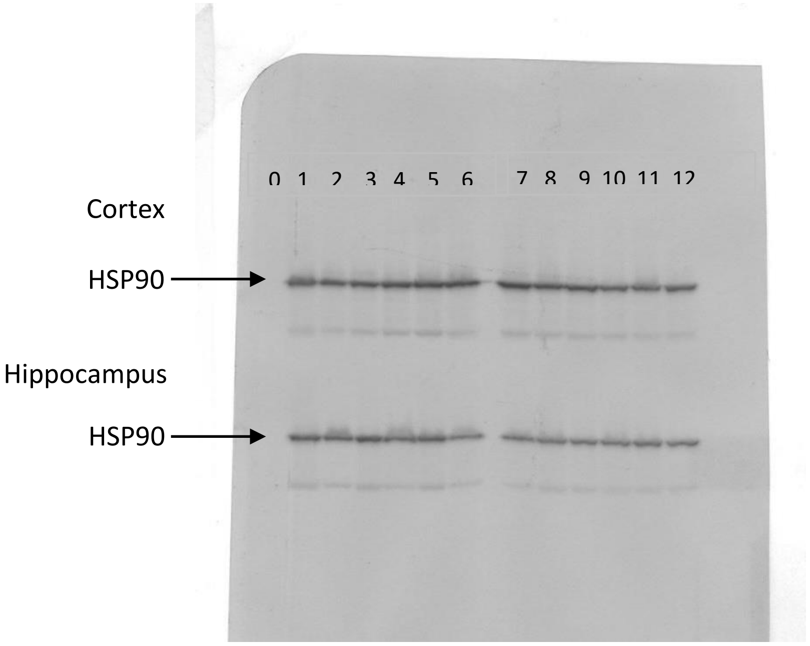

Legend: 0-Protein Ladder; 1-3 NaCl; 4-6 PCP; 7-9 NaCl-H; 10-12 PCP-H

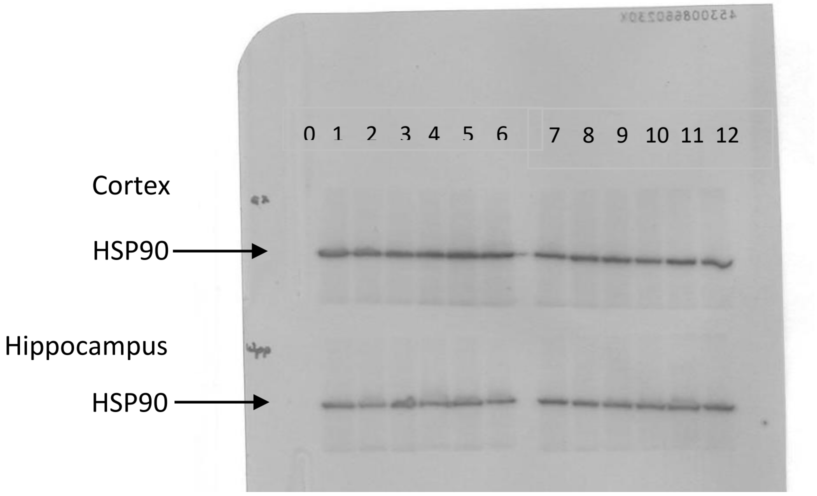

Legend: 0-Protein Ladder; 1-3 NaCl; 4-6 PCP; 7-9 NaCl-C; 10-12 PCP-C

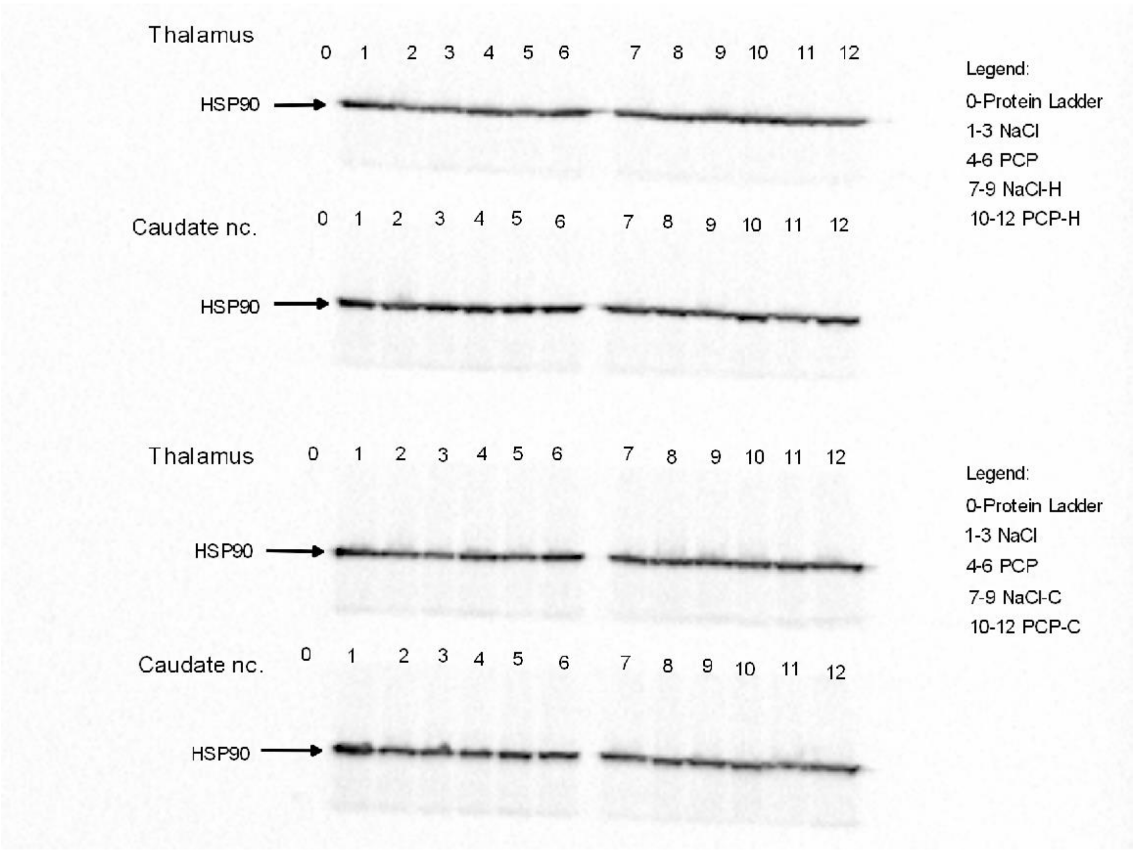
Thalamus
0 1 2 3 4 5 6 7 8 9 10 11 12
HSP90
Caudate nc.
0 1 2 3 4 5 6 7 8 9 10 11 12
HSP90
Legend:
0-Protein Ladder
1-3 NaCl
4-6 PCP
7-9 NaCl-H
10-12 PCP-H
Thalamus
0 1 2 3 4 5 6 7 8 9 10 11 12
HSP90
Caudate nc.
0 1 2 3 4 5 6 7 8 9 10 11 12
HSP90
Legend:
0-Protein Ladder
1-3 NaCl
4-6 PCP
7-9 NaCl-C
10-12 PCP-C

FKBP51

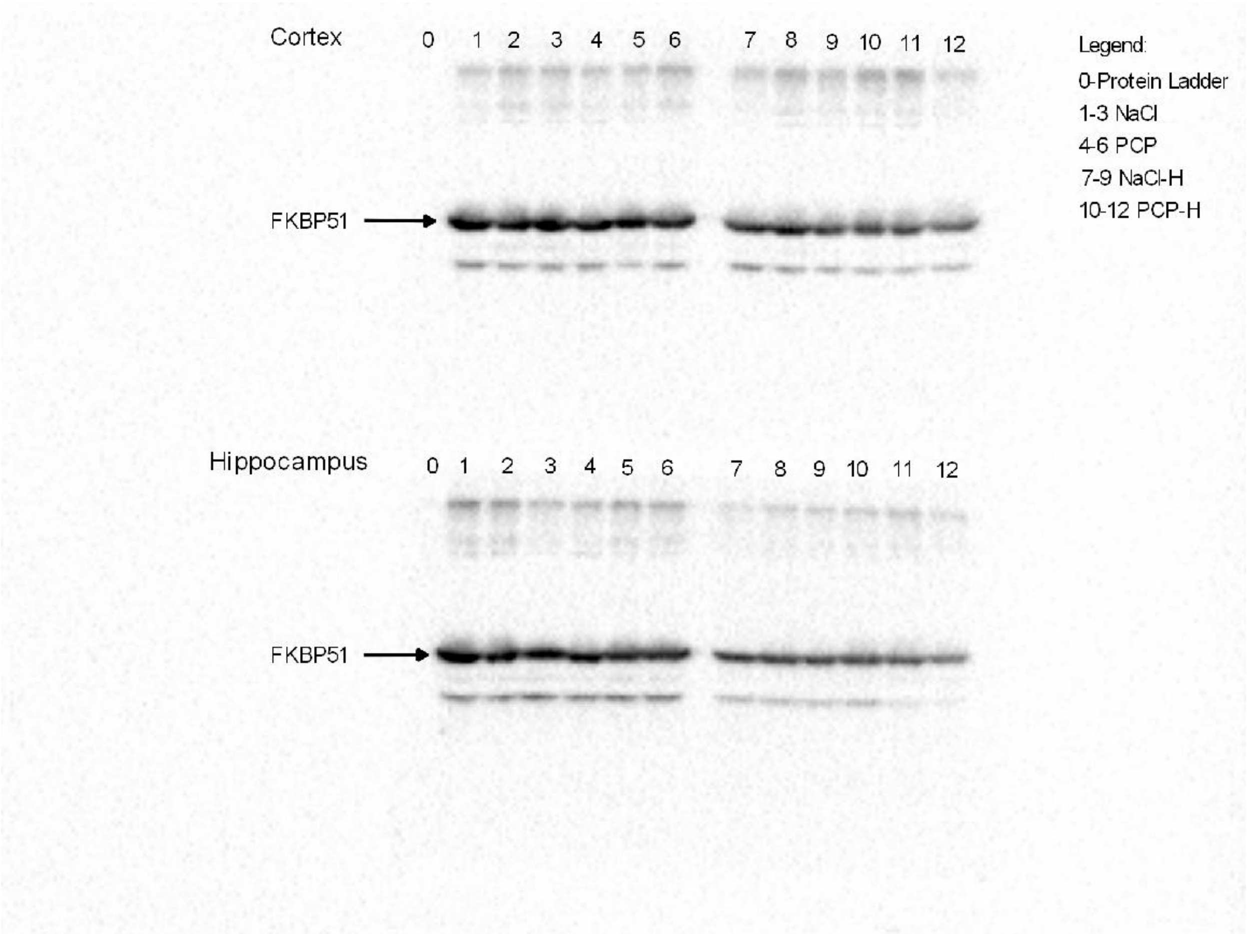
Cortex
0 1 2 3 4 5 6 7 8 9 10 11 12
Legend:
0-Protein Ladder
1-3 NaCl
4-6 PCP
7-9 NaCl-H
10-12 PCP-H
FKBP51
Hippocampus
0 1 2 3 4 5 6 7 8 9 10 11 12
FKBP51

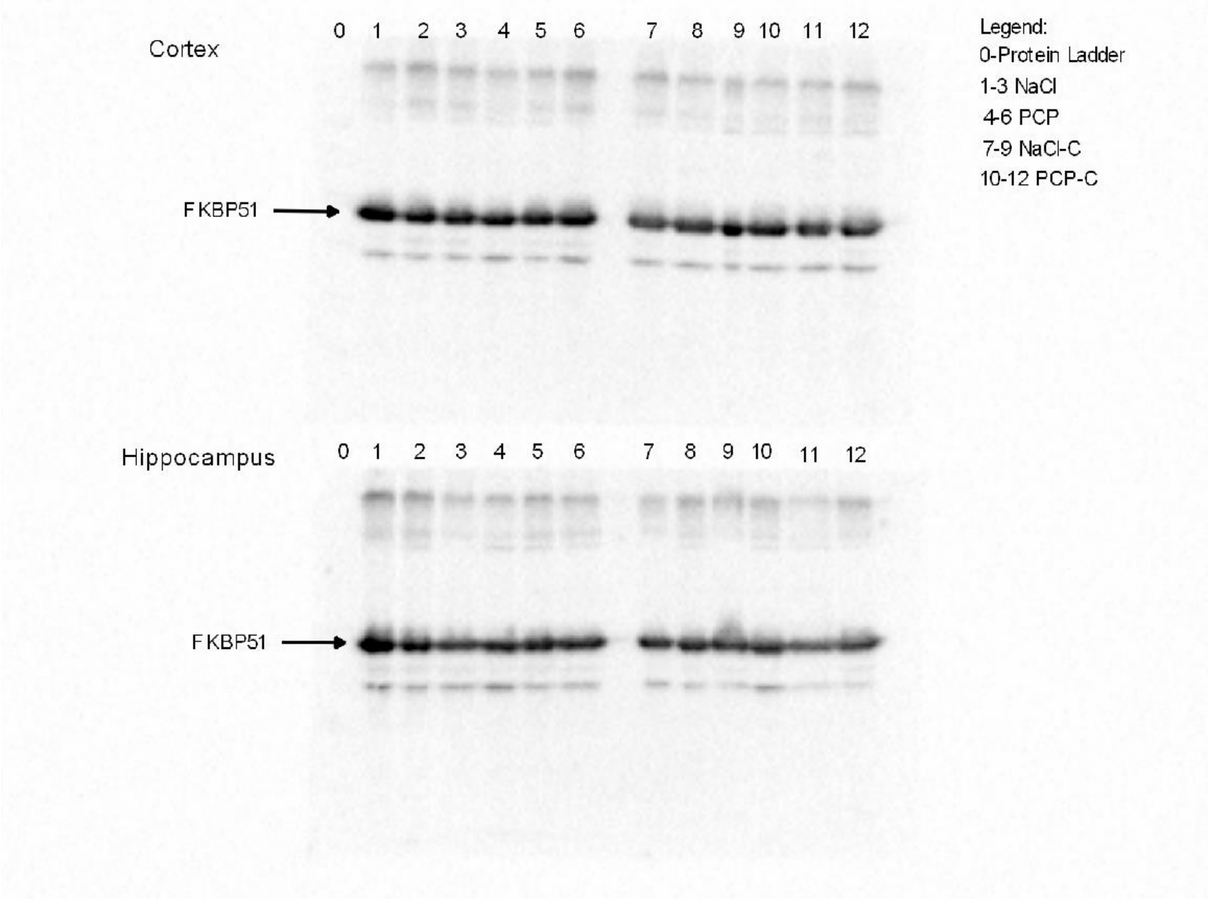
Cortex
0 1 2 3 4 5 6 7 8 9 10 11 12
Legend:
0-Protein Ladder
1-3 NaCl
4-6 PCP
7-9 NaCl-C
10-12 PCP-C
FKBP51
Hippocampus
0 1 2 3 4 5 6 7 8 9 10 11 12
FKBP51

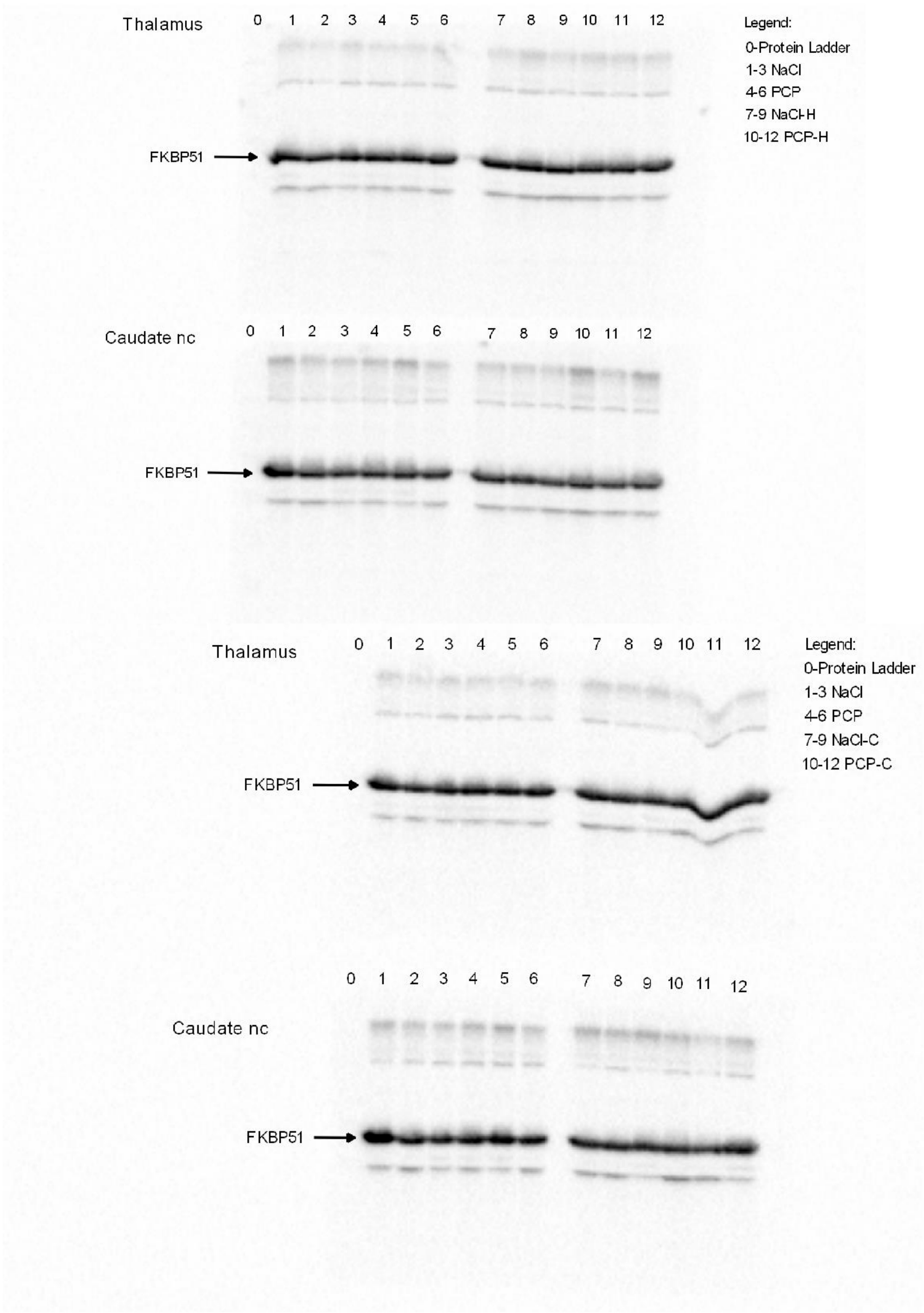
Thalamus
0 1 2 3 4 5 6 7 8 9 10 11 12
Legend:
0-Protein Ladder
1-3 NaCl
4-6 PCP
7-9 NaCl-H
10-12 PCP-H
FKBP51
Caudate nc
0 1 2 3 4 5 6 7 8 9 10 11 12
FKBP51
Thalamus
0 1 2 3 4 5 6 7 8 9 10 11 12
Legend:
0-Protein Ladder
1-3 NaCl
4-6 PCP
7-9 NaCl-C
10-12 PCP-C
FKBP51
0 1 2 3 4 5 6 7 8 9 10 11 12
Caudate nc
FKBP51

11β-HSD1

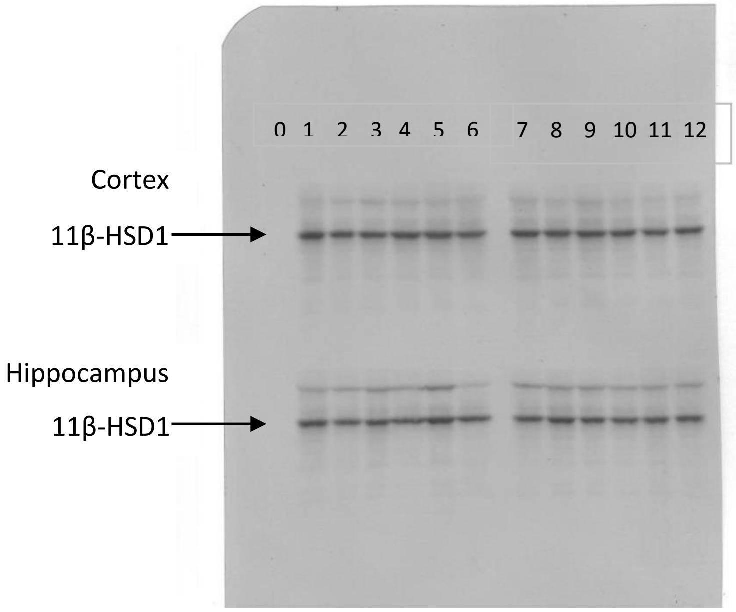

Legend: 0-Protein Ladder; 1-3 NaCl; 4-6 PCP; 7-9 NaCl-H; 10-12 PCP-H

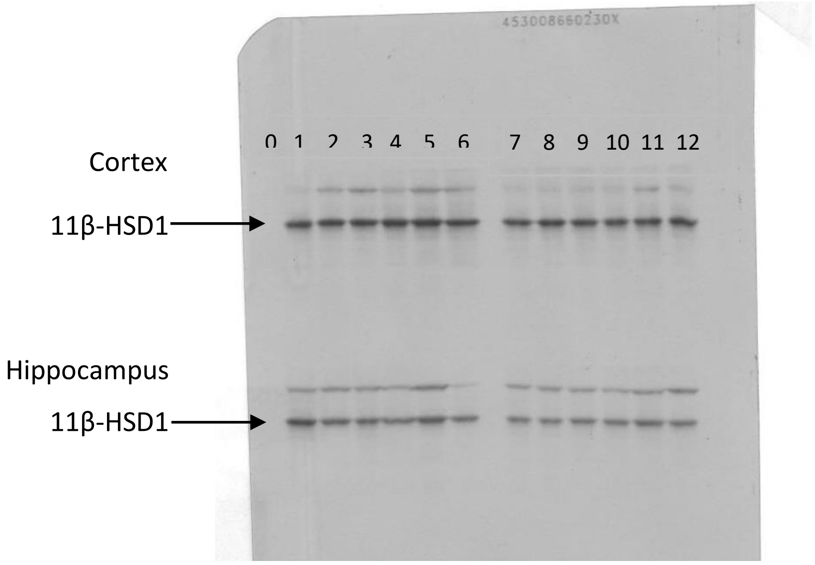

Legend: 0-Protein Ladder; 1-3 NaCl; 4-6 PCP; 7-9 NaCl-C; 10-12 PCP-C

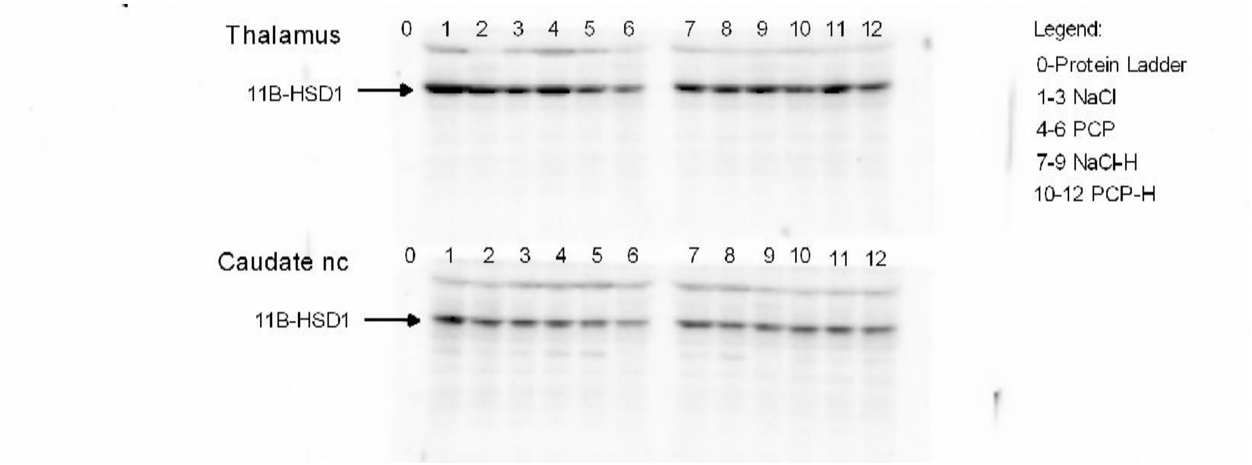
Thalamus
0 1 2 3 4 5 6 7 8 9 10 11 12
11B-HSD1
Caudate nc
0 1 2 3 4 5 6 7 8 9 10 11 12
11B-HSD1
Legend:
0-Protein Ladder
1-3 NaCl
4-6 PCP
7-9 NaCl-H
10-12 PCP-H

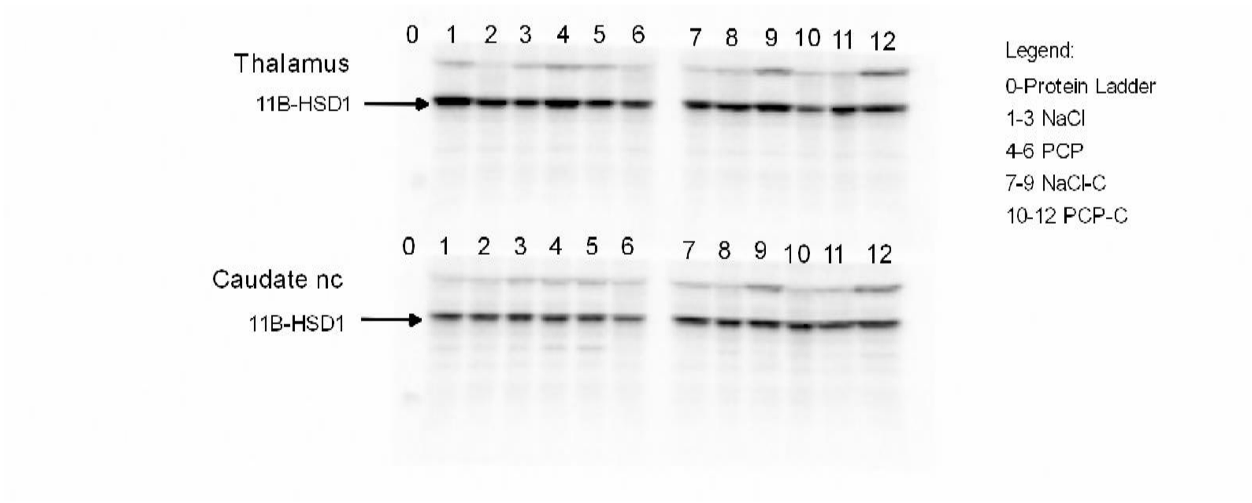
Thalamus
0 1 2 3 4 5 6 7 8 9 10 11 12
11B-HSD1
Caudate nc
0 1 2 3 4 5 6 7 8 9 10 11 12
11B-HSD1
Legend:
0-Protein Ladder
1-3 NaCl
4-6 PCP
7-9 NaCl-C
10-12 PCP-C

Actin

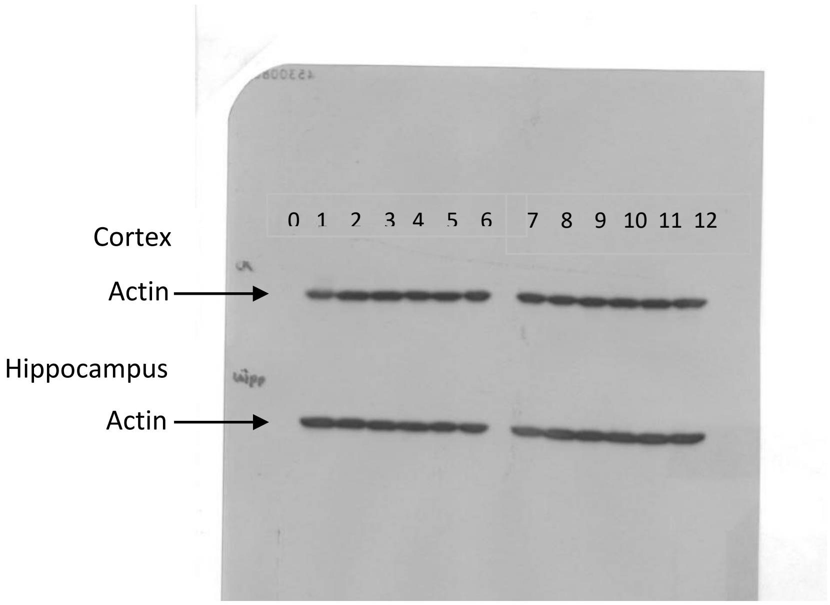

Legend: 0-Protein Ladder; 1-3 NaCl; 4-6 PCP; 7-9 NaCl-H; 10-12 PCP-H

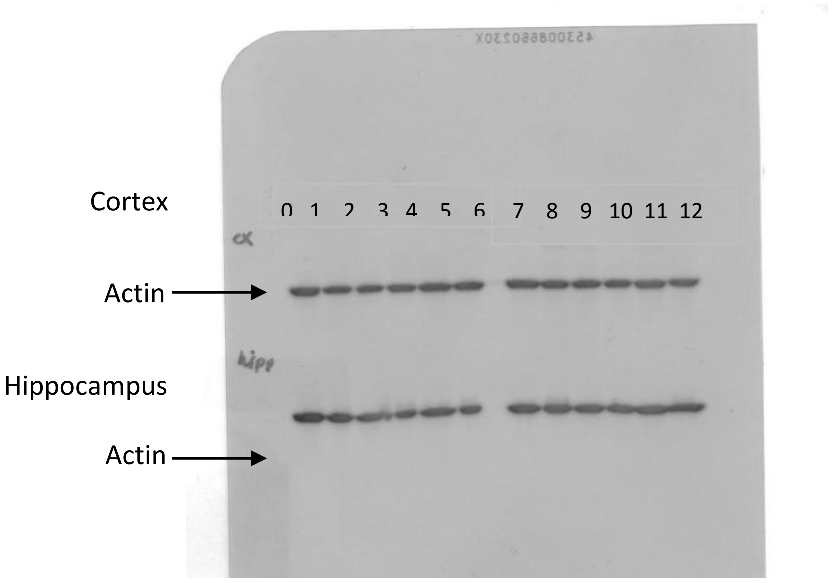

Legend: 0-Protein Ladder; 1-3 NaCl; 4-6 PCP; 7-9 NaCl-C; 10-12 PCP-C

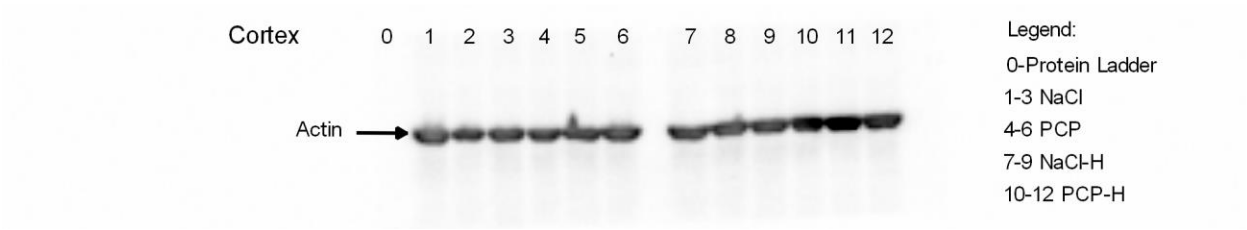
Cortex
0 1 2 3 4 5 6 7 8 9 10 11 12
Actin
Legend:
0-Protein Ladder
1-3 NaCl
4-6 PCP
7-9 NaCl-H
10-12 PCP-H

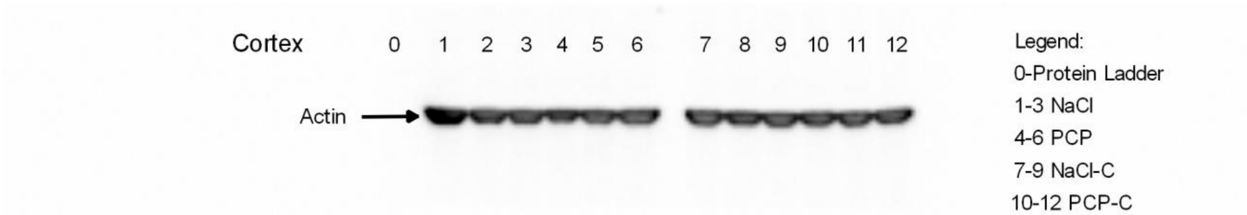
Cortex
0 1 2 3 4 5 6 7 8 9 10 11 12
Actin
Legend:
0-Protein Ladder
1-3 NaCl
4-6 PCP
7-9 NaCl-C
10-12 PCP-C

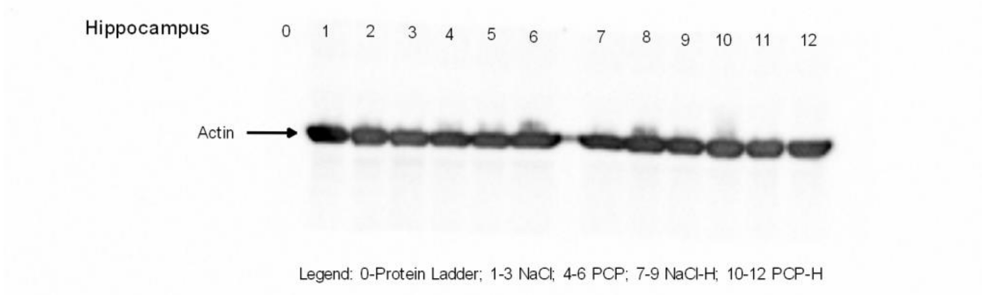
Hippocampus
0 1 2 3 4 5 6 7 8 9 10 11 12
Actin
Legend: 0-Protein Ladder; 1-3 NaCl; 4-6 PCP; 7-9 NaCl-H; 10-12 PCP-H

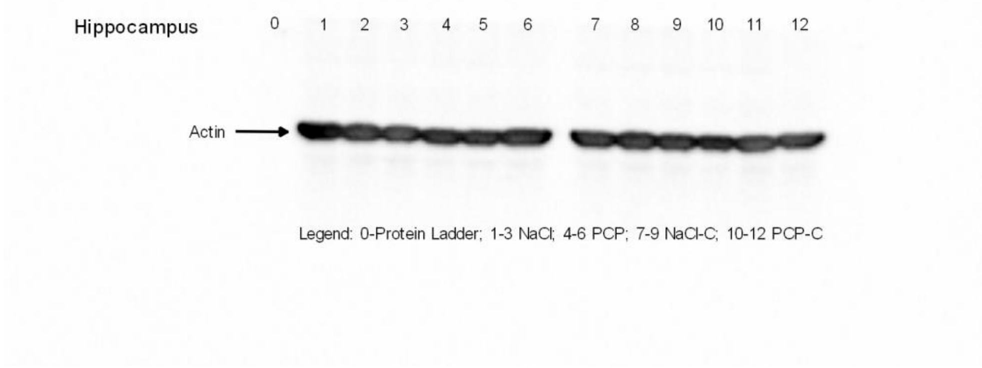
Hippocampus
0 1 2 3 4 5 6 7 8 9 10 11 12
Actin
Legend: 0-Protein Ladder; 1-3 NaCl; 4-6 PCP; 7-9 NaCl-C; 10-12 PCP-C

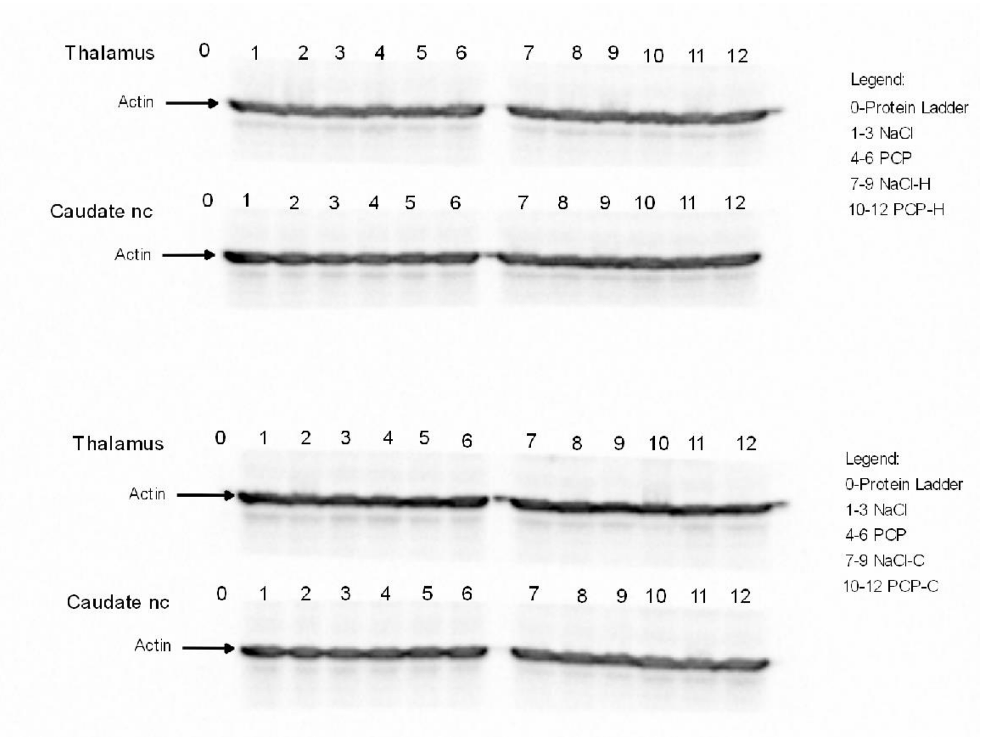
Thalamus
0 1 2 3 4 5 6 7 8 9 10 11 12
Actin
Caudate nc
0 1 2 3 4 5 6 7 8 9 10 11 12
Actin
Legend:
0-Protein Ladder
1-3 NaCl
4-6 PCP
7-9 NaCl-H
10-12 PCP-H
Thalamus
0 1 2 3 4 5 6 7 8 9 10 11 12
Actin
Caudate nc
0 1 2 3 4 5 6 7 8 9 10 11 12
Actin
Legend:
0-Protein Ladder
1-3 NaCl
4-6 PCP
7-9 NaCl-C
10-12 PCP-C
